# Putative modulation of the gut microbiome by probiotics enhances preference for novelty in a preliminary double-blind placebo-controlled study in ferrets

**DOI:** 10.1186/s42523-020-00030-y

**Published:** 2020-05-05

**Authors:** Supritha Dugyala, Travis S. Ptacek, Jeremy M. Simon, Yuhui Li, Flavio Fröhlich

**Affiliations:** 1 Department of Biology, University of North Carolina at Chapel Hill, Chapel Hill, NC 27599 USA; 2 UNC Neuroscience Center, University of North Carolina, Chapel Hill, NC USA; 3 Carolina Institute for Developmental Disabilities, University of North Carolina, Chapel Hill, NC USA; 4 Department of Genetics, University of North Carolina, Chapel Hill, NC USA; 5 Department of Psychiatry, University of North Carolina at Chapel Hill, Chapel Hill, NC 27599 USA; 6 Carolina Center for Neurostimulation, University of North Carolina at Chapel Hill, Chapel Hill, NC 27599 USA; 7 Department of Neurology, University of North Carolina at Chapel Hill, Chapel Hill, NC 27599 USA; 8 Department of Biomedical Engineering, University of North Carolina at Chapel Hill, Chapel Hill, NC 27599 USA; 9 Department of Cell Biology and Physiology, University of North Carolina at Chapel Hill, 115 Mason Farm Rd. NRB 4109F, Chapel Hill, NC 27599 USA

**Keywords:** 16S rRNA community profiling, Autism Spectrum Disorder., *Bifidobacterium infantis.*, Butyrate., Fecal Microbiome., Ferret (*Mustela putorius furo*)., Probiotic Intervention., Short Chain Fatty Acids.

## Abstract

**Background:**

Increasing evidence suggests a causal relationship between the gut microbiome and psychiatric illnesses. In particular, autism spectrum disorder is associated with gastrointestinal symptoms and alterations in the gut microbiome. Administration of probiotics is a commonly used strategy by caregivers of people with neurodevelopmental illness. However, evidence for successful improvement in gut microbiome and (behavioral) symptoms has been lacking.

**Results:**

Here, we use a novel ferret model of maternal immune activation to show that high-dose probiotic administration in a placebo-controlled study design causes changes in the gut microbiome in the form of a transient increase in the administered bacterial species. In contrast, we found no differences in baseline microbiome composition or changes induced by probiotic administration between animals exposed in utero to maternal immune activation and control animals. However, the relative presence of several bacterial species correlated with an increased preference for novelty (object and conspecific). Intriguingly, several of the hits in this screen are species that have previously emerged in the literature as being associated with autism and anxiety.

**Conclusions:**

Together, our results suggest that high-dose probiotic interventions may be beneficial for the adjunct treatment of psychiatric illnesses. Placebo-controlled clinical trials in humans are urgently needed.

## Introduction

There is growing evidence for a causal relationship between the composition of the gut microbiome and behavior. One of the most striking examples is that neurodevelopmental disorders appear to be related to the physiology of the gastrointestinal tract. A well-documented aspect of this link is the prevalence of gastrointestinal symptoms in individuals with neurodevelopmental disorders. For example, children with autism spectrum disorder (ASD) often experience gastrointestinal distress [1]. Symptoms of abdominal pain, constipation, diarrhea, and bloating are common throughout childhood and may persist into adulthood in this patient population [2]. Interestingly, the severity of gastrointestinal symptoms appear to correlate with the severity of developmental symptoms in children with ASD [3]. The etiology of ASD and the symptoms associated with it remain unclear. However, it is worth noting that differences in naturally occurring bacterial colonies can be found in the microbiome of individuals with ASD when compared to typically developing individuals [1]. There is also evidence that malabsorption and maldigestion of certain macromolecules in children with ASD are correlated to changes in gut microbiota at the genus and species level [4].

Some of these microbiome changes have been replicated in animal models. For example, a recent study in a mouse model of ASD has demonstrated pathological changes in gastrointestinal physiology and microbial composition [5]. In this model, maternal immune activation (MIA) during pregnancy (at a time point corresponding to the second trimester in humans) is used as an early perturbation to the developmental trajectory [6]. Intriguingly, administration of healthy bacteria (“probiotics”) to juvenile MIA mice in order to supplement their original gut microbiota composition was successful in not only partially restoring gastrointestinal health but also alleviating several of the ASD-like behavioral phenotypes [5]. This study demonstrated a general dysbiosis in the gut microbiota in juvenile MIA mice as well as the ability to modulate the gut microbiome by supplementing with probiotics. Despite this, it has remained unclear if probiotic interventions have long term effects and whether they modulate the gut ecosystem and behavior in the adult offspring in the MIA model. To address this gap, we performed a double-blind placebo-controlled probiotic intervention study in adult ferrets in our recently developed ferret MIA model [7]. Ferrets share important features of structure and function of the central nervous system with humans [8, 9] and have emerged as promising, cost-effect intermediate model system for translational neuroscience studies [10].

## Methods and materials

### Animals

Twenty adult (6 months of age) male ferrets (*Mustela putorius furo*) were used in this study. These animals were housed separately on a 12-h light-dark cycle. Animals had ad libitum access to food and water. All animals were fed with Teklad 2072 ferret diet (Envigo, Indianapolis, IN). The manufacturer lists the following average nutrient profile for this diet: 39% protein, 19% fat, 1.2% crude fiber. All animal procedures were performed in compliance with the National Institutes of Health guide for the care and use of laboratory animals (NIH Publications no. 8023, revised 1978), and approved by the Institutional Animal Care and Use Committee of the University of North Carolina at Chapel Hill and the United States Department of Agriculture (USDA Animal Welfare).

The adult ferrets used in this study were born to 6 pregnant ferrets in house. As previously described [7], at G30, i.e., day 30 of gestation, the pregnant mother animals were randomly assigned to receive either 10 mg/kg PolyIC (polyinosinic:polycytidylic acid, potassium salt, Sigma-Aldrich, St. Louis, MO, dissolved 10 mg/ml in PBS) or 1 ml/kg PBS via intraperitoneal (i.p.) injection under anesthesia introduced by 4–5% then sustained by 1–2% isoflurane. Immune activation was confirmed by elevated rectal temperature measurements taken immediately before and 3 hours after the PBS or PolyIC injection. Blood samples (0.5 ml) were also drawn from the jugular vein at the same time points. Serum cytokine level (IL1β, IL-2, IL-6, INF-γ and TNFα) was then detected by the UNC Animal Clinical Chemistry and Gene Expression Laboratory using multiplexed biomarker immunoassays (Luminex MAGPIX system, Luminex Inc., Austin, TX, using the canine cytokine kit). Ferret offspring (kits) were born at around G40-G41. The kits were kept with their mother until weaning at postnatal day 42. At this time, the male ferrets were separated for single housing [7].

### Probiotic intervention

The probiotics used in this study was VSL #3 (Alpha Sigma, Covington, LA), a mixture of 8 strains of lactic acid–producing bacteria (*L. plantarum, L. delbrueckii subsp. Bulgaricus, L. casei, L. acidophilus, Bifidobacterium breve, B. longum, B. infantis*, and *Streptococcus salivarius subsp. thermophilus*) [11]. We chose to use VSL#3 since it includes several different gram-positive strains as we had no a priori hypothesis for which strain would be the most beneficial in the context of our study. VSL#3 has been investigated in several other animal models such as dogs [12] and rodents [13–17]. Thus, further motivation for the choice of probiotic brand was to build on these previous studies. The VSL#3 was shipped in a cooler with cool packs and was immediately refrigerated at arrival until preparation for administration. The VSL#3 suspension was prepared daily by diluting 4.5 × 10^9^ colony-forming units (CFU) of freeze-dried VSL#3 in 10 ml Kitty Milk Replacement (KMR).

### Study schedule

All ferrets received 10 ml of KMR with probiotics or KMR alone every day in the evening for 6 consecutive days. All animals were also placed on 12 h of water restriction per day during this time. Weight variations and changes in health condition were monitored and none occurred. Water restriction before administration of the probiotic mixture helped the ferrets more easily accept 10 ml of liquid without the discomfort. The 10 ml of fluid were fed to the animal with a syringe without needle. Care was taken that the animal swallowed the fluid. All adult ferrets (10 each from the PBS or PolyI:C condition) were randomly chosen to receive either the probiotic mixture or KMR alone, creating 4 groups (Probiotic:PolyI:C, Probiotic:PBS, KMR;PolyI:C, KMR:PBS).

The researchers involved with the administration of the interventions and behavioral assays were blinded to the experimental condition of all animals. This included both the identity of the animal pertaining to their assignment to the MIA or control (PBS) group as well as assignment to probiotics or control (KMR).

### Microbiome sample collection and sequencing

Stool collections took place in the housing facilities of the animals. Stool samples were taken 3 times. Collection time points are labeled with letters A, B, and C throughout the manuscript. Collection A was performed 22 days before the first day of probiotic treatment as a baseline. Collection B was performed 2 days after the probiotic treatment concluded in order to avoid recording a predictably high number of bacteria sourced from the probiotic itself. Collection C occurred 9 days after the probiotic treatment concluded.

On each collection day, each single house cage was cleared of enrichment materials and litter boxes and disinfected with Vimoba 128 (Quip Laboratories, Wilmington, DE). Each sample was collected within 5 min of deposit and immediately frozen in a dry ice bath (−80^o^ C) before further processing. The 16S rDNA amplicon sequencing was performed at the University of North Carolina Microbiome Core Facility (Chapel Hill, NC) as previously described [7]. Briefly, DNA was extracted from stool samples using MagMAX Total Nucleic Acid Isolation kit (Thermo Fisher Scientific, Waltham, MA). From this DNA the V3-V4 region of the bacterial 16S rRNA gene was amplified. This PCR product was purified and then used to generate a sequencing library for sequencing on an Illumina MiSeq instrument.

### Behavioral assessments

Behavioral tests were performed around the time-point of the first sample collection (Time-point A) and after sample collection at Time Point C. No behavioral assessments were performed at Timepoint B since the goal of the study was to investigate behavioral changes after completion of a course of probiotic administration. All behavioral tests were performed in a well-illuminated white 1.5 X 1.5 m^2^ arena as previously described [7]. The arena was cleaned with 70% ethanol between tests on different animals, and the tests were conducted by experimenters blind to the group membership of the animals. The activity within the box was captured by a top-mounted camera as previously described (Microsoft LifeCam Cinema 720p HD Webcam, 30 Hz frame rate) [7].

The *novel object recognition* test is widely used to investigate recognition memory without the need for behavioral training or conditioning [18]. It has been used to demonstrate behavioral changes in rodents that are akin to ASD symptoms [5, 19, 20]. In summary, the animal was first placed in the arena for acclimation for 3 min. In the next Learning Phase, the animal was removed while 2 identical objects were placed in opposite corners of the arena allowing for 15 cm of space between the object and the walls. The animal was replaced into the arena and allowed to interact with the objects for 5 min. Two and a half hours passed before the recall phase began. In this phase, one of the identical objects was swapped with a similarly sized and weighted novel object. The animal was placed back into the arena and interactions with either object were recorded for 5 min. Recognition of the novel object is characterized as the time the animal spends interacting with the novel object minus the time spent interacting with the old one. The two types of objects used were green and black 10 lb. kettle bells with ferret toys attached to them. The identity of the novel object and the novel object location were randomized and balanced across animals of the two treatment groups.

**Fig. 1:**
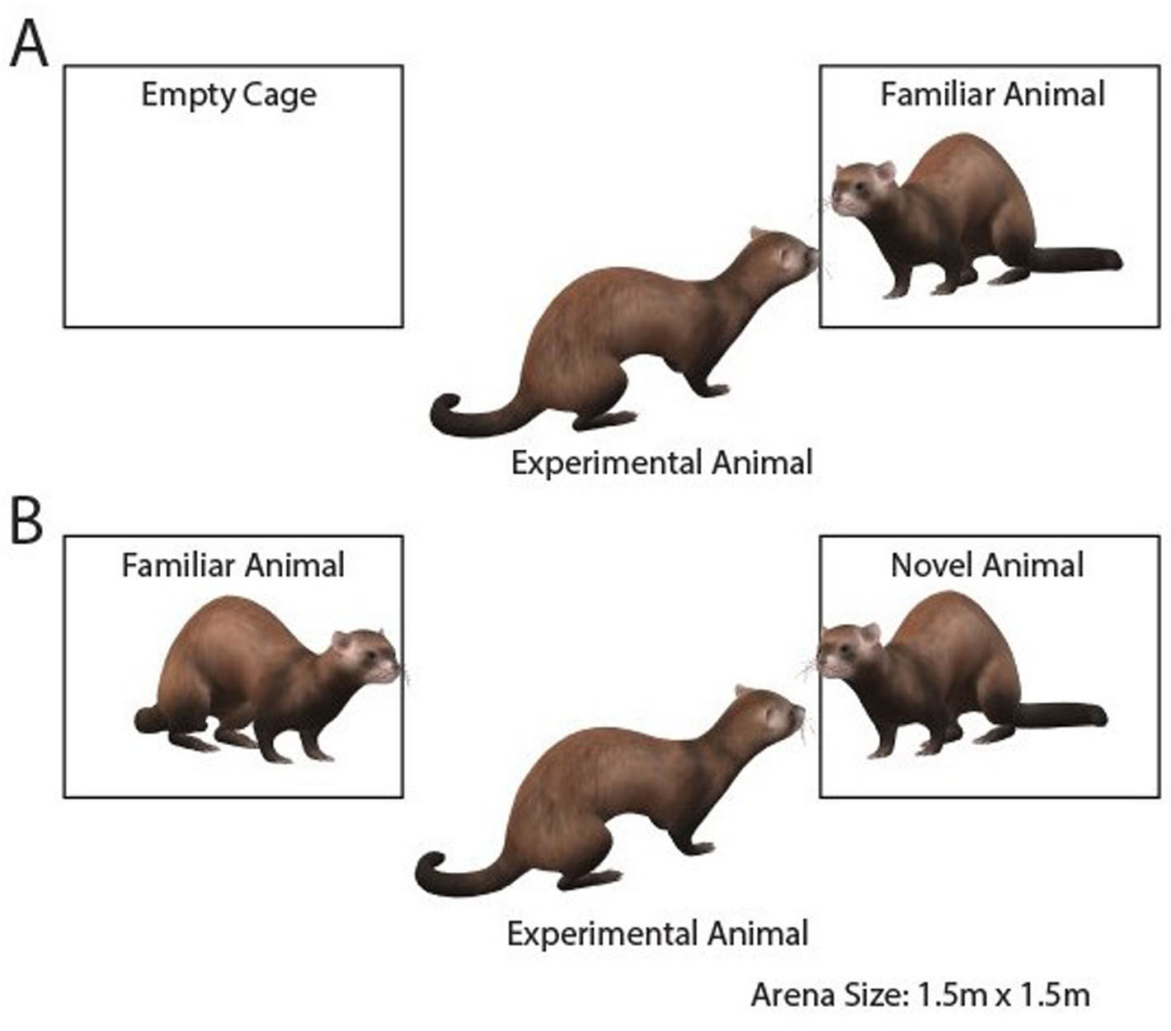
Picture of the social interaction behavioral test, showing the experimental animal in 2 phases of the paradigm. **a** depicts the animal socializing with a control animal which will become the familiar animal in (**b)**, where the experimental animal can choose to socialize with either the familiar animal from the previous phase or a novel ‘stranger’ animal

This social interaction test has historically been used to investigate how animals interact with strange and familiar conspecifics. A three chamber paradigm test has been successfully utilized in rodent studies to observe social affiliation and social memory [21–23]. This test is also used to measure ASD related behavioral changes [5, 24]. In the ferret version of this task, two identical cages were placed within the arena 15 cm away from the walls. The cages are 49 × 33 × 26 cm^3^ (L x W x H) and have windows at all four sides and were weighed down with 10 lb. weights (Fig. 1). The animal was placed into the arena with the empty cages to acclimate for 10 min before the next phase. Once the test animal was removed from the arena, a “stranger” animal of identical sex that was not utilized in this study was placed in one of the cages. The test animal was replaced in the arena for 10 min. The sociability of the animal is determined by subtracting the amount of time the animal spends interacting with the empty cage from the amount of time it spends interacting with the cage containing the stranger. Following this phase, another stranger was placed into the other cage. The familiar and stranger animal locations on either side of the arena were randomized across the study. The interactions of the test animal were recorded again for 10 min. The social preference of the animal is characterized by subtracting the time spent interacting with the familiar animal from the time spent interacting with the stranger. All cages and the surrounding arena were cleaned with 70% ethanol between tests.

### Behavioral data analysis

BORIS software [25] was used to manually log behavioral events in novel object recognition and social interaction test as previously described [7]. BORIS enables frame-by-frame analysis of a video and allows recording of the duration of interaction in each assay. In analyzing the novel object recognition video, we logged the time periods during which the animal actively explored either of the objects by pointing their noise towards the object. For the social interaction videos, we logged the timing when the animal explored either of the cages in a similar way.

### Microbiome analysis

Initial analysis of the microbiome sequencing data, including quality control of the fastq files, quantification of operational taxonomical units (OTUs), and calculation of alpha and beta diversity, was performed as previously described [7]. Briefly, fastq files were quality filtered for reads with > 70% of bases having a quality score > 24 using FastQC. QIIME [26] was used to join quality filtered fastqs and align them to the GreenGenes 13 05 reference of 16S rRNA sequences, to pick and quantify OTUs (UCLUST), and calculate alpha and beta diversity (samples rarefied at depth of 1000 for alpha diversity). An empty sample was included as a control for reagent contamination. This sample generated only 136 reads and did not have any identifiable OTUs, ruling out any significant reagent contamination. Differences in groups by beta diversity were tested using QIIME’s implementation of the PERMANOVA test (*p* < 0.05 considered significant).

To determine whether OTU relative frequency related to animal behavior, we performed spearman correlations of OTU relative frequency at time point C with percent time novel object minus percent time at familiar object and with percent time at novel anima minus percent time at familiar animal. To determine whether the relative frequencies of OTUs and of OTUs representing probiotic organisms (genera Bifidobacterium, Lactobacillus and Streptococcus, annotated as k__Bacteria;p__Actinobacteria;c__Actinobacteria;o__Bifidobacteriales;f__Bifidobacteriaceae;g__Bifidobacterium, k__Bacteria;p__Firmicutes;c__Bacilli;o__Lactobacillales;f__Lactobacillaceae;g__Lactobacillus, and k__Bacteria;p__Firmicutes;c__Bacilli;o__Lactobacillales;f__Streptococcaceae;g__Streptococcus, respectively) were related, we performed Spearman correlations between the relative frequency of OTUs at time point C and the relative frequencies of probiotic OTUs at time point B. We used time point B for probiotic OTUs, as we found that probiotic OTU frequency in treated animals tended to peak at time point B. OTUs with a frequency of zero in more than 10 animals (half of all animals) were excluded from correlation analyses to avoid spurious correlation results.

To identify OTUs that may connect probiotic treatment with changes in behavior, we filtered for OTUs that were correlated with either time at novel animal or time at novel object and with at least one of the three probiotic organism OTUs. For all correlation tests, we filtered for a correlation coefficient (Spearman’s rho) greater than 0.3 and a p value less than 0.1. Although a p value less than 0.05 is generally considered to be statistically significant, we chose the higher p value threshold to capture correlations that were trending significance. All correlation tests and plots were generated in R. Correlation tests of significance were performed using Algorithm AS 89 as implemented in cor.test(). Plots of probiotic quantities at the three time points were generated using the ggplot2 package using the loess method to smooth the curves.

## Results

The overall goal of this study was to investigate if a probiotic intervention alters the gut microbiome in ferrets and whether such changes alter behavior. We focused our investigation on the novel object and novel animal assays since they have obvious conceptual parallels with symptoms in ASD [23] and also showed robust deficits when compared to health animals in our previous work [7]. The study comprised three interrelated questions: (1) Does probiotic supplementation modulate behavior? (2) Does probiotic supplementation alter the gut microbiome? (3) Do changes in gut microbiome correlate with changes in behavior?

### Does probiotic supplementation modulate behavior?

We tested whether probiotic treatment modulates performance in the novel object and novel animal assays. We first established if our previously published MIA intervention was successful in this current study by examining body temperature and cytokine levels of the ferrets pregnant with the offspring investigated here. To confirm that PolyIC injections triggered a maternal immune response, we measured the body temperature (Table 1) and elevation of maternal serum cytokine IL1-β, IL-2, IL-6, INF-γ, TNF-α (Table 2) three hours after injection. Elevation of serum concentration of these cytokines has been established in a mouse model [27] and in our previous ferret study [7]. Concentration of TNF-α was significantly higher in animals that received PolyIC injections compared to animals that received PBS (control) injection. Levels of IL-6 as well as body temperatures was trend-level higher in those animals that received PolyIC injections, as well (body temperature after injection: PolyIC = 39.10 ± 1.15 °C, n = 4; PBS = 37.90 ± 0.22 °C, n = 4, unpaired t test, t (9) = 2.05, p = 0.087). We note that the small number of pregnant ferrets used for this study did not suffice to have enough statistical power for this exploratory analysis.

**Table 1:** Average maternal body temperature 3 h after PolyIC or PBS injection

	PolyIC (*n* = 4)	PBS (*n* = 4)
Body Temperature (C °)	39.1 ± 1.15	37.9 ± 0.22 ^#^

^#^*t*_*(6)*_ = 2.05, *p* = 0.087, unpaired *t* test

Values are mean ± SD

**Table 2:** Cytokine concentration before and 3 h after PolyIC or PBS injection in mother animals of the ferrets investigated in this study

	Cytokine concentration (pg/ml)			
	PolyIC (*n* = 4)		PBS (*n* = 4)	
	Before	After	Before	After
IL1-β	16.6 ± 8	24.2 ± 30.2	7.9 ± 13.7	10.2 ± 18
IL-2	5.3 ± 8.6	111.8 ± 166.1	6.6 ± 7.6	5.6 ± 6.4
IL-6	15 ± 14	116.7 ± 127.2	9.2 ± 8	7.9 ± 6.9 #
INF-γ	46 ± 72.7	10.5 ± 1.7	14.5 ± 21.3	14.6 ± 21.3
TNF-α	7.8 ± 6.7	164.9 ± 151.2	6.1 ± 7	4.9 ± 5.8 *

^#^*t*_*(6)*_ = 2.36, *p* = 0.057, unpaired *t* test

^*^t_*(6)*_ = 2.58, *p* = 0.042

Values are mean ± SD

MIA and control animals were treated with probiotics dissolved in milk or milk without probiotics in a double-blind placebo-controlled study design. The behavioral tests were performed before and after the 6 days of probiotics administration. After collecting behavioral data from the 20 animals, each video was coded for interaction with either the novel object or novel animal in the arena. Quantitative values were assigned to interactions based on the percent of time spent with the familiar object or animal subtracted from the time spent with the novel object or animal. We did not find evidence for differences in change in behavior when comparing the results from before and after the intervention between the four groups (Table 3). We hypothesized that the variability of response to probiotic treatment, combined with the relatively low number of animals in our study was obscuring the effects of probiotics on behavior. Because of this, we next examined the change in microbiome and its relationship between behavior (from the post intervention behavioral analysis) and composition of the microbiome regardless of maternal exposure (PolyIC or PBS) group.

**Table 3:** Behavioral changes by probiotic intervention among four different experiment groups

		Difference Pre vs Post		ANOVA	
		Mean	Std Dev	Test	*P*-value
**Novel Object**					(*F*-value)
Poly IC	Milk	−0.2	38.8	Probiotic	0.88 (0.02)
Poly IC	Probiotic	12.1	37.0	Poly IC	0.53 (0.04)
PBS	Milk	−0.1	41.9	Interaction	0.53 (0.41)
PBS	Probiotic	−7.6	11.4		
**Novel Animal**					
Poly IC	Milk	−33.6	15.9	Probiotic	0.17 (2.11)
Poly IC	Probiotic	55.2	82.7	Poly IC	0.71 (0.14)
PBS	Milk	3.4	71.6	Interaction	0.11 (2.85)
PBS	Probiotic	−3.3	61.5		
**Animal vs Empty**					
Poly IC	Milk	4.3	32.5	Probiotic	0.17 (2.06)
Poly IC	Probiotic	−27.8	43.9	Poly IC	0.82 (0.05)
PBS	Milk	−9.2	11.9	Interaction	0.54 (0.04)
PBS	Probiotic	−21.6	41.1		

Sample size = 5 for each experiment group

In all ANOVA tests, df1 is 1 for each factor, df2 is 19

### Does probiotic supplementation alter the gut microbiome?

**Fig. 2:**
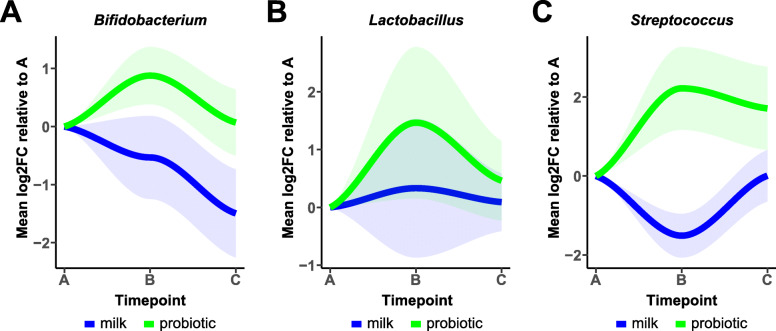
Change in relative frequency of probiotic organisms over time. The log2 fold change in relative frequency of Bifidobacterium, Lactobacillus, and Streptococcus (panels **a**, **b** and **c**, respectively) relative to timepoint A is plotted over timepoints A, B and C. Average relative frequency in animals treated with probiotics is plotted in green. Average relative frequency in animals treated with milk alone is plotted in blue. Shaded area around the line indicates +/− one standard error

We first tested grouping of samples by probiotic treatment and maternal PolyI:C exposure after characterizing and quantifying OTUs in fecal samples. There was no significant difference in beta diversity between animals with maternal PolyI:C exposure and controls prior to probiotic (PERMANOVA p > 0.05, Table 4), and in agreement with the behavioral analyses, there was no significant difference in beta diversity after treatment between animals grouped by probiotic treatment or maternal PolyI:C exposure (PERMANOVA p > 0.05, Table 4). These results were consistent with our failure to find evidence for differences in behavioral performance as a function of either probiotic treatment and PolyI:C exposure. We thus collapsed the data across PolyI:C exposure to investigate if there were changes in the microbiome associated with probiotic administration when compared to placebo. To determine when probiotics may have their greatest effect on the gut microbiome, we performed an exploratory analysis by plotting the average probiotic organism frequency across the three time points (Fig. 2). All three probiotic organisms peaked at time point B and then fell back to approximately baseline at time point C. The normalized increase in relative frequency for the probiotic group (green) was numerically larger than for the control (KMR, blue) group. These findings suggest that probiotic administration caused an increased in certain species.

**Table 4:** Microbiome beta diversity

		PERMANOVA p-value by clustering method	
Comparison	Timepoint	unweighted unifrac	weighted unifrac
PBS vs PIC	A	0.407	0.811
Prob vs Milk in All	B	0.67	0.123
	C	0.904	0.835
Prob vs Milk in PBS	B	0.792	0.371
	C	0.985	0.654
Prob vs Milk in PIC	B	0.281	0.157
	C	0.884	0.674

### Do changes in gut microbiome correlate with changes in behavior?

**Fig. 3:**
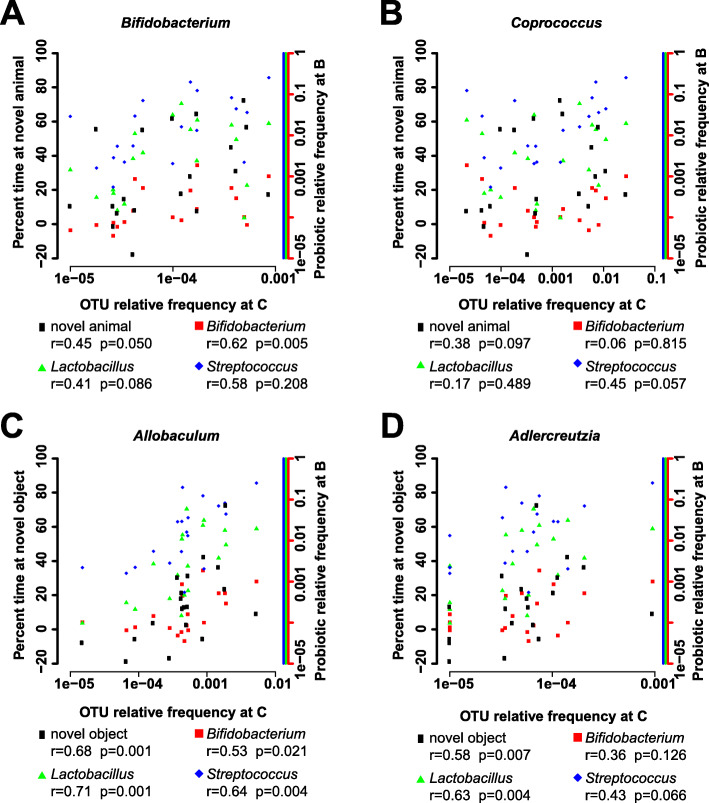
Correlation of OTUs with novel animal or novel object behavior and with probiotic organism relative frequency. Plots are shown for the OTUs *Bifidobacterium*, *Coprococcus*, *Allobaculum* and *Aldercreutzia* in panels **a, b, c** and **d**, respectively. Relative OTU frequency at timepoint C is plotted on the x axis. Percent time at novel object or novel animal, minus percent time at familiar object or familiar animal, is plotted on the left y axis, and relative frequency of probiotic organisms at timepoint B is plotted on the right y axis. The left y axis and x axis are plotted in log10 scale. OTU relative frequency / novel object/animal pairs are plotted in black rectangles, while OTU relative frequency / probiotic organism relative frequency pairs are plotted in red squares, green triangles and blue diamonds (for *Bifidobacterium*, *Lactobacillus*, and *Streptococcus*, respectively). The Spearman rho (r) and nominal p-value for each correlation is indicated in the key for each plot

Due to the lack of significant changes in the microbiome by treatement group, we decided to correlate OTU frequency with probiotic organism frequency and behavior data to more directly test whether probiotic treatment could be connected to behavioral changes via changes in the microbiome. In fecal samples from the 20 animals, we identified 273 OTUs at the genus level. Of these 273, 101 had a frequency greater than 0 in at least half of all animals and were therefore used for correlation analysis. Probiotic organism frequency at timepoint B was used for correlation analysis as our prior results showed probiotic organism frequency peaking at B (Fig. 2). OTU frequency at time point C was used, as this time point was closest to when the behavioral data after the intervention were collected. The goal of this analysis was to identify OTUs that exhibited a persistent modulation by probiotic treatment that also had effects on behavior. This analysis allows for the identification of potential causal links between probiotic treatment and behavioral performance in the presence of high inter-individual compositional variability of the intestinal microbiome. Given our filtering thresholds for correlation (Spearman’s Rho > 0.3, p < 0.1), we identified six OTUs (time point C) correlated with percent time at novel object and six other OTUs (time point C) correlated with percent time at novel animal that were also correlated with at least one probiotic organism at time point B (Table 5). An OTU for one of the probiotic organisms itself, *Bifidobacterium* was correlated with novel animal behavior. Several of these OTUs had been previously reported to be associated with autism and anxiety phenotypes. Plots for these OTUs (OTU frequency at time point C versus behavior and time point B probiotic organism frequency) are shown in Fig. 3. All 273 OTUs identified, and their full correlation results are found in Supplementary Table 1. Together, these results suggest that changes in composition of the gut microbiome are related to the two novelty behaviors examined in our study.

**Table 5:** Correlation of OTUs and novelty behavior

	Behavior		Bifidobacterium		Lactobacillus		Streptococcus	
	coeff	p-val	coeff	p-val	coeff	p-val	coeff	p-val
**Novel Object Correlations**								
k__Bacteria;p__Actinobacteria;c__Coriobacteriia;o__Coriobacteriales;f__Coriobacteriaceae;g__	0.411	0.072	0.121	0.621	0.626	0.004	0.489	0.034
k__Bacteria;p__Actinobacteria;c__Coriobacteriia;o__Coriobacteriales;f__Coriobacteriaceae;g__Adlercreutzia	0.582	0.007	0.364	0.126	0.633	0.004	0.430	0.066
k__Bacteria;p__Firmicutes;c__Clostridia;o__Clostridiales;f__Ruminococcaceae;g__	0.483	0.033	0.196	0.418	0.512	0.027	0.418	0.077
k__Bacteria;p__Firmicutes;c__Clostridia;o__Clostridiales;f__[Mogibacteriaceae];g__	0.618	0.004	0.184	0.452	0.236	0.331	0.572	0.010
k__Bacteria;p__Firmicutes;c__Erysipelotrichi;o__Erysipelotrichales;f__Erysipelotrichaceae;g__Allobaculum	0.680	0.001	0.532	0.021	0.705	0.001	0.637	0.004
k__Bacteria;p__TM7;c__TM7–3;o__CW040;f__F16;g__	−0.516	0.020	−0.083	0.734	−0.445	0.056	−0.287	0.233
**Novel Animal Correlations**								
k__Bacteria;p__Actinobacteria;c__Actinobacteria;o__Bifidobacteriales;f__Bifidobacteriaceae;g__Bifidobacterium	0.447	0.050	0.621	0.005	0.405	0.086	0.582	0.010
k__Bacteria;p__Bacteroidetes;c__Bacteroidia;o__Bacteroidales;f__S24–7;g__	0.402	0.080	0.170	0.485	0.281	0.243	0.500	0.031
k__Bacteria;p__Bacteroidetes;c__Flavobacteriia;o__Flavobacteriales;f__Flavobacteriaceae;g__Capnocytophaga	0.456	0.045	0.135	0.580	0.160	0.512	0.519	0.024
k__Bacteria;p__Firmicutes;c__Bacilli;o__Lactobacillales;f__Carnobacteriaceae;g__Carnobacterium	0.382	0.097	0.056	0.820	0.211	0.385	0.440	0.061
k__Bacteria;p__Firmicutes;c__Clostridia;o__Clostridiales;f__Lachnospiraceae;g__Coprococcus	0.382	0.097	0.058	0.815	0.168	0.489	0.446	0.057
k__Bacteria;p__Proteobacteria;c__Gammaproteobacteria;o__Pasteurellales;f__Pasteurellaceae;g__	−0.494	0.027	0.255	0.292	0.513	0.025	0.303	0.208

coeff = Spearman’s Rho for correlation of behavior/probiotic (time point C) with OTU (time point B)

p-val = p-value of correlation by algorithm AS 89

## Discussion

In this study we were able to identify bacterial genera that putatively link reduction of an autism-like phenotype in ferrets with frequency of probiotic organisms after treatment. Our original hypothesis that the behavior of MIA animals would be normalized by probiotic treatment was not supported at the treatment group level, as the behavioral tests in this study did not yield significant or trending-significant differences between animals that received probiotic intervention to those that did not. We also tested for differences in the microbiome between MIA animals and control animals at baseline, and within groups before and after treatment. Again, there were no significant differences detected at the treatment group level. Previously, we have reported baseline differences between MIA and control animals [28]. In the previous study, the effect was strongest in females and muted in males. The present study used only male animals, as the social behavioral abnormalities were more pronounced in males in our previous study of MIA in the ferret.

Although statistical power in our study was low due to a low number of animals, we hypothesized that variability in probiotic treatment effect and in animal behavior had also obscured the effects of probiotic treatment, and that significant effects might still be observable by looking directly at phenotype and probiotic organism quantification rather than treatment groups. By correlating OTU frequency at the later timepoint with the frequency of probiotic organisms at the earlier timepoint where probiotic organism frequency (and presumably, the effects of these organisms on the microbiome) peaked, we identified organisms associated with stable long-term effects of probiotic treatment. Overlapping this group of organisms with OTUs having frequencies at the later timepoint correlated with behavior at the same timepoint gives us a set of organisms that putatively modulate behavior as a result of on probiotic treatment. We performed this analysis on all 20 animals regardless of treatment group, as the effects of probiotic treatment and effects on behavior were more precisely measured by direct correlation with peak probiotic organism frequency and the numeric measure of behavioral phenotypes. We identified 12 different bacterial genera with frequencies at the time of testing that were correlated both with one of these behavioral phenotypes, and with the frequency of at least one of the three probiotic organisms at peak frequency. For correlation testing we filtered for organisms with a Spearman’s Rho > 0.3 and a nominal *p* < 0.1. This correlation threshold is modest, and the p value threshold is both greater than the common *p* < 0.05 threshold for significance and does not include correction for multiple comparisons. The purpose of using these thresholds was to identify organisms trending toward significance, as our study was underpowered for statistical significance. These organisms should therefore be considered putative targets. However, several of these genera have been implicated in the modulation of behavior in past studies, which supports the plausibility of our findings. In our data set, the frequency of *Allobaculum*, *Aldercreutzia*, and *Coprococcus* were all positively correlated with time at either novel object or novel animal and were associated with the frequency of at least one of the probiotic organisms two days after treatment. *Allobaculum* [29] and *Aldercreutzia* (or its family *Coriobacteriaceae*) [30–32] have been reported to be negatively correlated with anxiety. *Coprococcus* has been found to be reduced in the microbiome of children with autism [33]. These organisms, therefore, are positively correlated with both a phenotype of increased preference for novelty in our ferret study and with lower symptoms of anxiety and ASD in published studies. We also show, for the first time, that these organisms are also positively correlated with probiotic organism frequency after treatment a month prior. This suggests a possible mechanistic connection between probiotic treatment and rescuing of these phenotypes.

Our study demonstrates how probiotics can have both direct and indirect effects on behavioral phenotypes. Probiotics can indirectly have effects on behavior by changing the gut microbiome through altering the microenvironment in the gut. VLS #3, the probiotic mixture utilized in this study, has been studied in the past as treatment for many diseases including ulcerative colitis, irritable bowel syndrome, and inflammatory bowel disease [34–36]. Some of these studies show changes in brain function and behavior as well [37]. In some models of stress, VSL #3 has been shown to alter microbiota composition in a way that changes gene expression in relation to pain transmission and inflammation in early life intervention [34]. VSL #3 has also been shown to improve social exploratory behaviors in rodent models of liver [38].

Some studies find that similar intervention utilizing different live strains of bacteria can cause temporary reorganization of the microbial environment. This reorganization can be correlated with different behavioral phenotypes like reduced anxiety-like behavior in rodent models after intervention with different strains of *Lactobacillus* [39]. In the case of both *Bifidobacterium* and *Lactobacillus*, an acidification of the surrounding environment causes an indirect shift in equilibrium between prevalence of different microbiota populations [39].

We were able to see putative indirect effects of probiotic treatment on behavior via several genera positively correlated with probiotic organisms at time of treatment and with behavior. This suggests probiotics change the gut microenvironment to promote these genera, and these genera then affect behavior.

We also observed direct effects of the probiotic organisms on behavior. One of the genera associated with probiotic organism frequency at treatment and behavior was one of the probiotic organisms itself. *Bifidobacterium* at timepoint C was correlated with novel animal behavior, and with *Bifidobacterium* after treatment (timepoint B). The correlation of a probiotic organism with itself at time of treatment is not a given: *Lactobacillus* and *Streptococcus* in the late timepoint (C) were not correlated with themselves or any other probiotic organism at time of treatment. One study highlights the direct behavioral effects of *Bifidobacterium infantis*, one of the strains used in this study, on Germ Free (GF) mice [40]. GF mice that were raised sterile were found to have a reduced stress response after gut reconstitution with the *Bifidobacterium*. This normalization of a heightened stress responses in GF mice was demonstrated again in further studies [41]. In conceptual analogy to these findings, a study in human patients with ASD reported a species of *Bifidobacterium* as one strain that was scarce in the gut microbiome [42]. A reduction in *Bifidobacterium* abundance in patients with ASD has also been exemplified in further studies [43].

A common mechanistic connection between several of the genera correlated with probiotic organisms and with behavior is the production of short chain fatty acids (SCFAs). SCFAs are byproducts of bacterial metabolism [44] that, in some cases have been shown to cross the blood brain barrier and affect behavioral phenotypes, like ASD. The effects on behavior depend on the SCFA. Two SCFAs, Butyrate (BA) and propionic acid (PPA), have been shown to have opposing effects in the developing brain, with PPA exposure promoting gliosis and neuroinflamation and BA promoting neuronal differentiation (Naser et al., 2019 [45]). Indeed, intracerebroventricular injection of PPA in rats induces social abnormalities and is used as model of ASD (Shultz et al., 2014 [46]). Like PPA, BA has also been shown to modulate behavior; it has the ability to cross the blood brain barrier and enact physiological change in the brain by upregulating levels of neurotransmitters like glutamate, glutamine, and γ-aminobutyric acid (GABA) [47]. *Bifidobacteria* have been shown to play a role in gut homeostasis and host health, supporting the production of acetate and lactate, which are compounds that are converted into BA by other bacteria [48]. As previously discussed, *Bifidobacterium* is both a probiotic organism contained in the probiotic used here and was one of the major correlative links between treatment and behavior change in this study. Changes in population frequency of SCFA producers in ASD have been previously reported. In a valproic acid-induced mouse model of ASD, the population of *Bacteroidetes* was decreased while the population of *Firmicutes* was increased [49, 50]. Human studies have shown the opposite, with Bacteroidetes increasing and Firmicutes decreasing in ASD [34, 51]. The main metabolic end products for the phylum *Bacteroidetes* are acetate and PPA while BA is the end point of most bacteria of the *Firmicutes* phylum [52]. In our study, one of our most significant hits for organisms positively correlated with both probiotic organisms and social behavior was *Allobaculum*, a *Firmicute* and known BA producer [53]. Additionally, several other *Firmicutes* were significantly, positively correlated hits in our study, including *Coprococcus* and *Carnobacterium*. As previously discussed, changes in both *Allobaculum* and *Coprococcus* have been implicated in autism and anxiety. In conclusion, our present study agrees with the PPA rat model of ASD and prior human studies. These studies all point to a model where PPA, exogenous or produced by *Bacteroidetes*, is associated with ASD, while BA, produced by *Firmicutes* like *Allobaculum* and promoted by *Bifidobacterium*, is associated with symptom resolution. Our study adds to this, suggesting a connection between probiotic treatment and BA producing organisms. Prior studies using the mouse valproic acid model of ASD do not agree with this model, which may indicate either that the PPA rat model and MIA ferret model are closer to human ASD, or that valproic acid induced ASD has a distinct etiology with respect to the gut microbome.

As with any scientific study, our study has a series of limitations. The first limitation is the small sample size in our population. Although we used 20 animals, these were divided into four groups, making pairwise group comparisons challenging due to the low number of samples per group. This limitation is probably directly responsible for our failure to find any differences between treatment groups either at the level of behavior or at the level of microbiome beta diversity and our inability to find organisms correlations with probiotic organism frequency and behavioral phenotypes that were significant after multiple comparison testing. This lack of statistical power is almost certainly due to the inherent variability in behavioral studies and in microbiome composition. This limitation could be addressed in future studies by adding more animals. It could also be addressed in part by measuring probiotic organism quantity by culturing, rather than OTU frequency, as culture would capture only viable organisms while 16S microbiome analysis captures both live and dead cells. Despite these limitations, we were able to derive promising results that were both consistent with and added to previously published studies of probiotics and of the microbiome in autism/anxiety by looking directly at correlations between behavioral phenotypes and the frequency of microbiome genera. These findings support the importance of future studies with larger sample sizes and culture-based quantification of probiotic organisms.

In conclusion, our study suggests that probiotics alter the gut microbiome and that these changes are correlated with behavioral phenotype of two assays of interest in novelty in ferrets. Given the potential of the ferret for the study of perturbations such as maternal immune activation that are associated with neurodevelopmental disorders, our findings open a new avenue towards a holistic understanding of the etiology of psychiatric illnesses and potential new treatment strategies based on targeted modulation of the gut microbiome.

## Supplementary information

**Additional file 1.**

## Data Availability

The raw fastq files from sequencing are publicly available in SRA project PRJNA529463. Any and all other raw or derived data are available from the corresponding author on reasonable request.

## Abbreviations

ASD: Autism Spectrum Disorder
BA: Butyrate
CFU: Colony Forming Unit
GF: Germ Free
KMR: Kitty Milk Replacement
MIA: Maternal Immune Activation
OTU: Operational Taxonomical Unit
PBS: Phosphate Buffered Saline
PCR: Polymerase Chain Reaction
PPA: Propionic Acid
PolyI:C: Polyinosinic-polycytidylic acid
QIIME: Quantitative Insights Into Microbial Ecology (software)
rDNA: Ribosomal DNA (rRNA genes)
rRNA: Ribosomal RNA
SCFA: Short Chain Fatty Acid
VSL#3: Name of a commercially available probiotic (Alpha Sigma, Covington, LA)
