# Supplementary figures and images for: Putative modulation of the gut microbiome by probiotics enhances preference for novelty in a preliminary double-blind placebo-controlled study in ferrets
Source: Anim Microbiome. 2020 May 5;2:14. doi: 10.1186/s42523-020-00030-y (PMC7266289; doi:10.1186/s42523-020-00030-y)

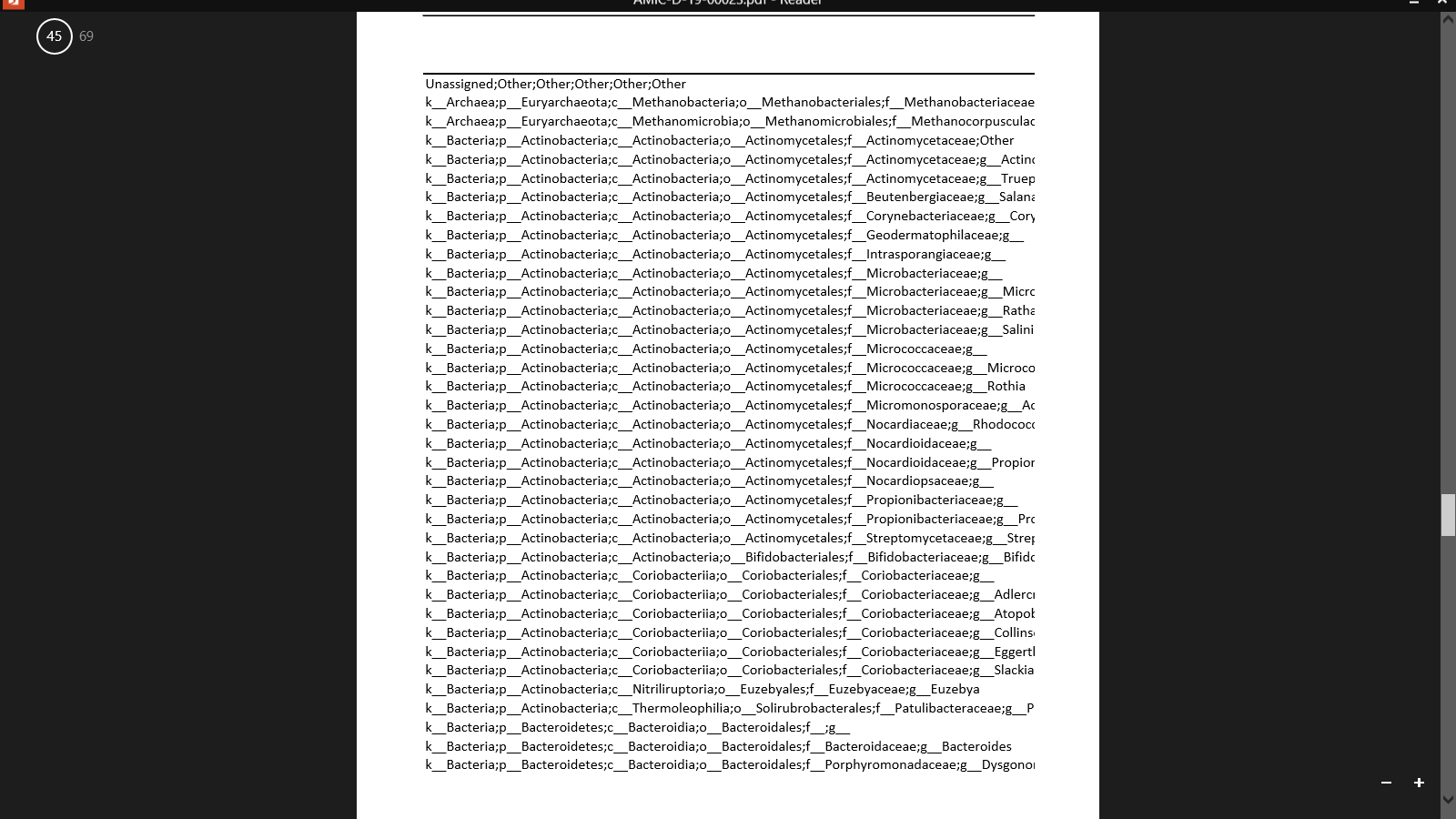


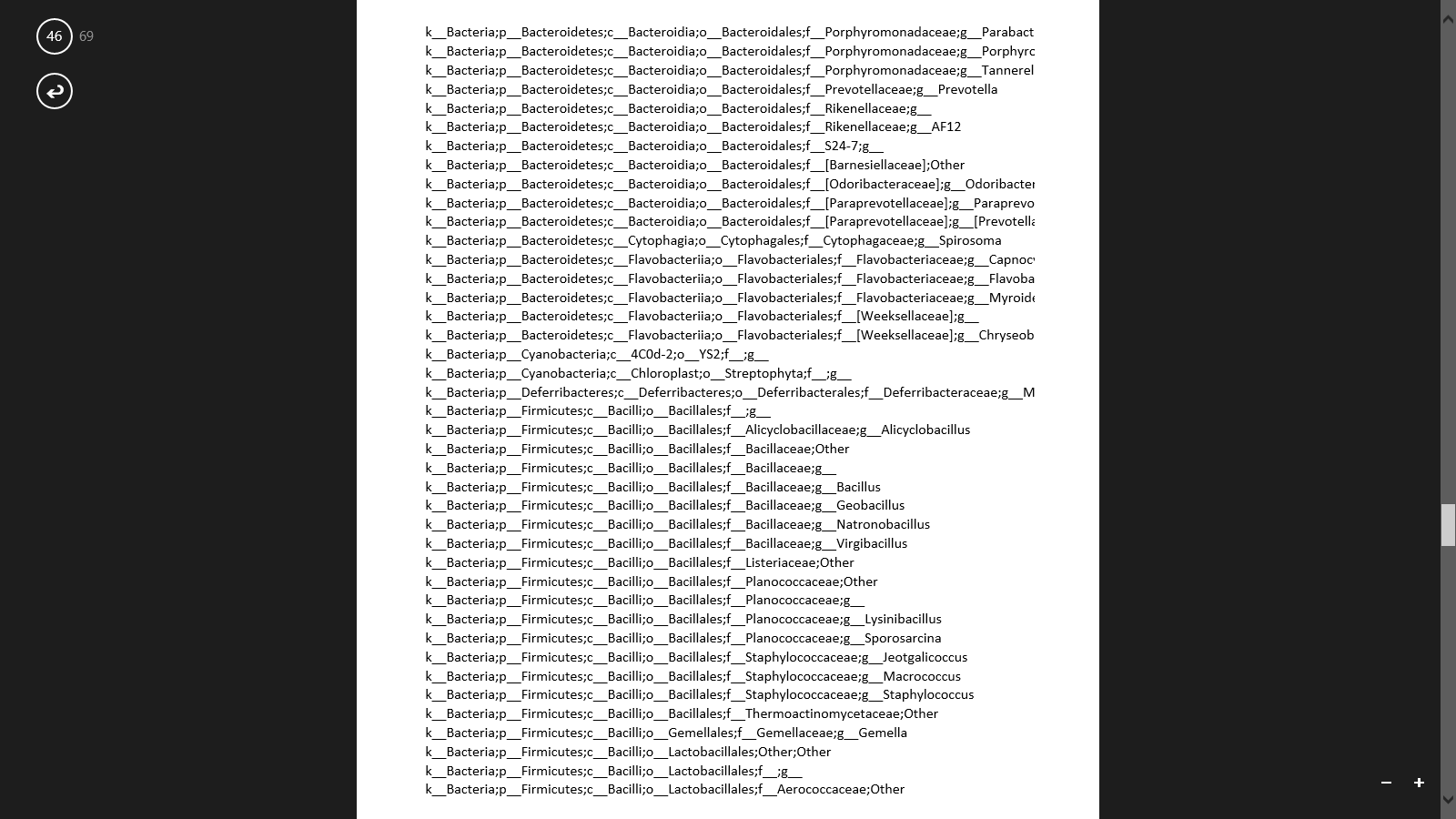


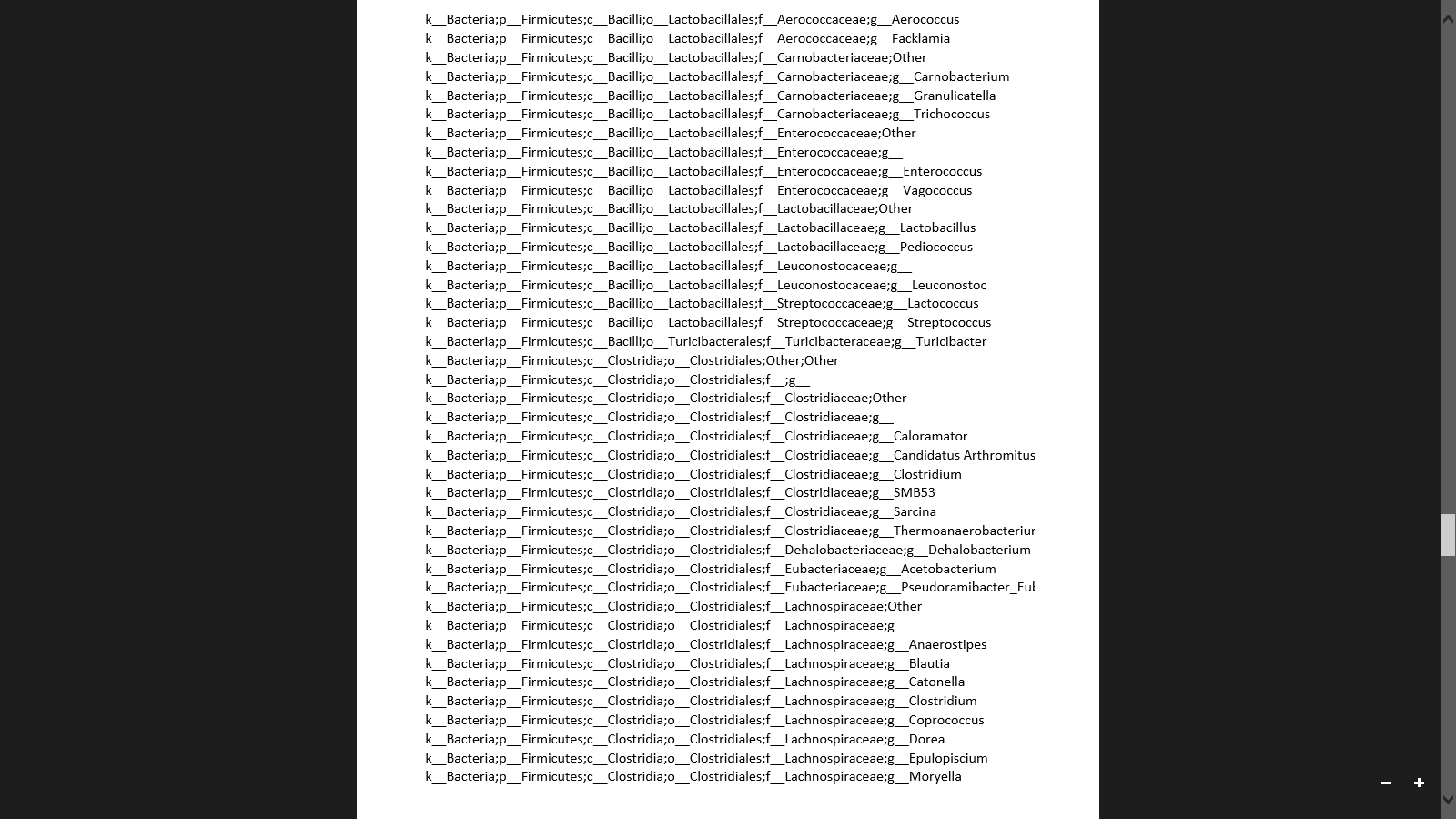


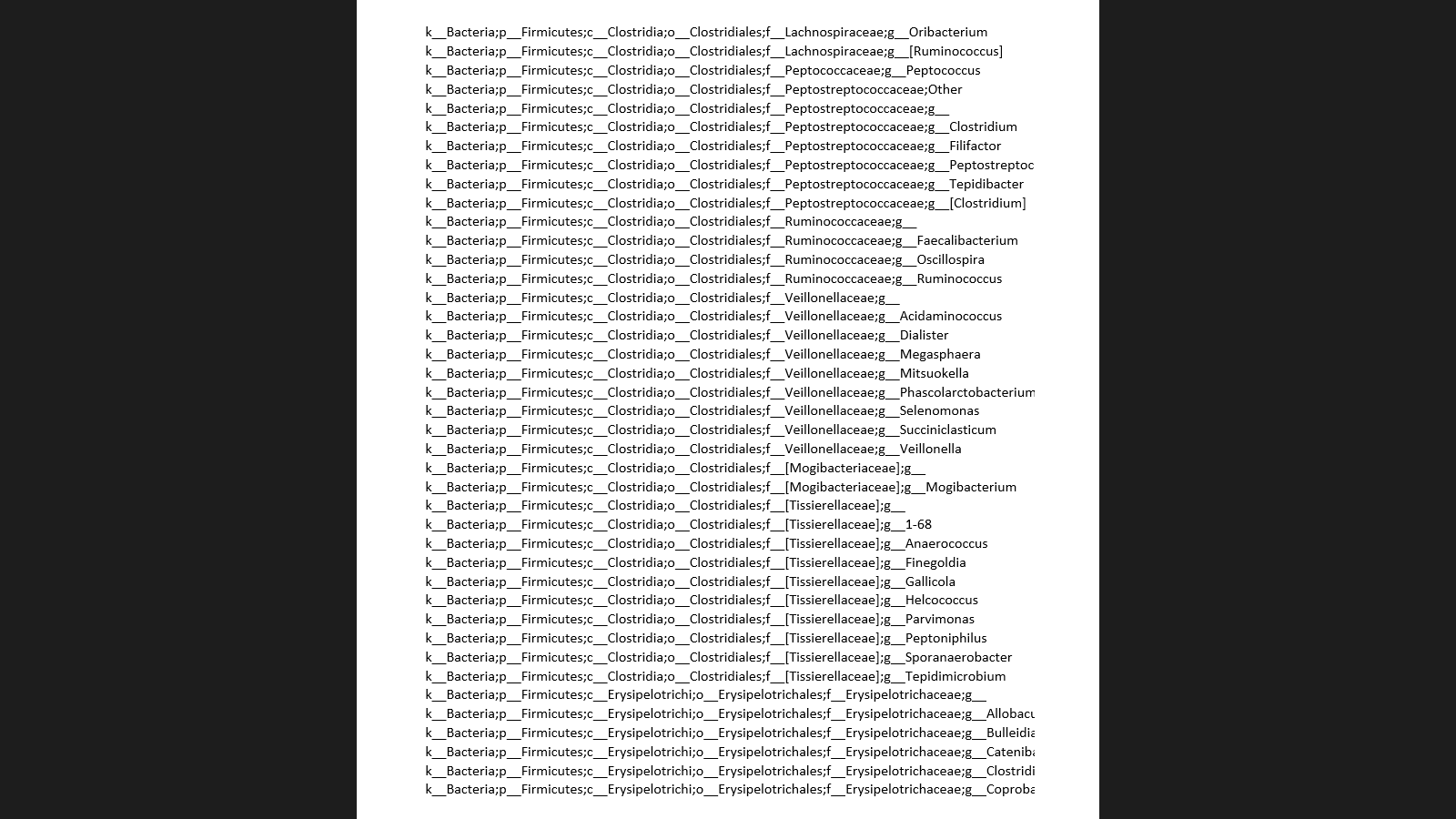


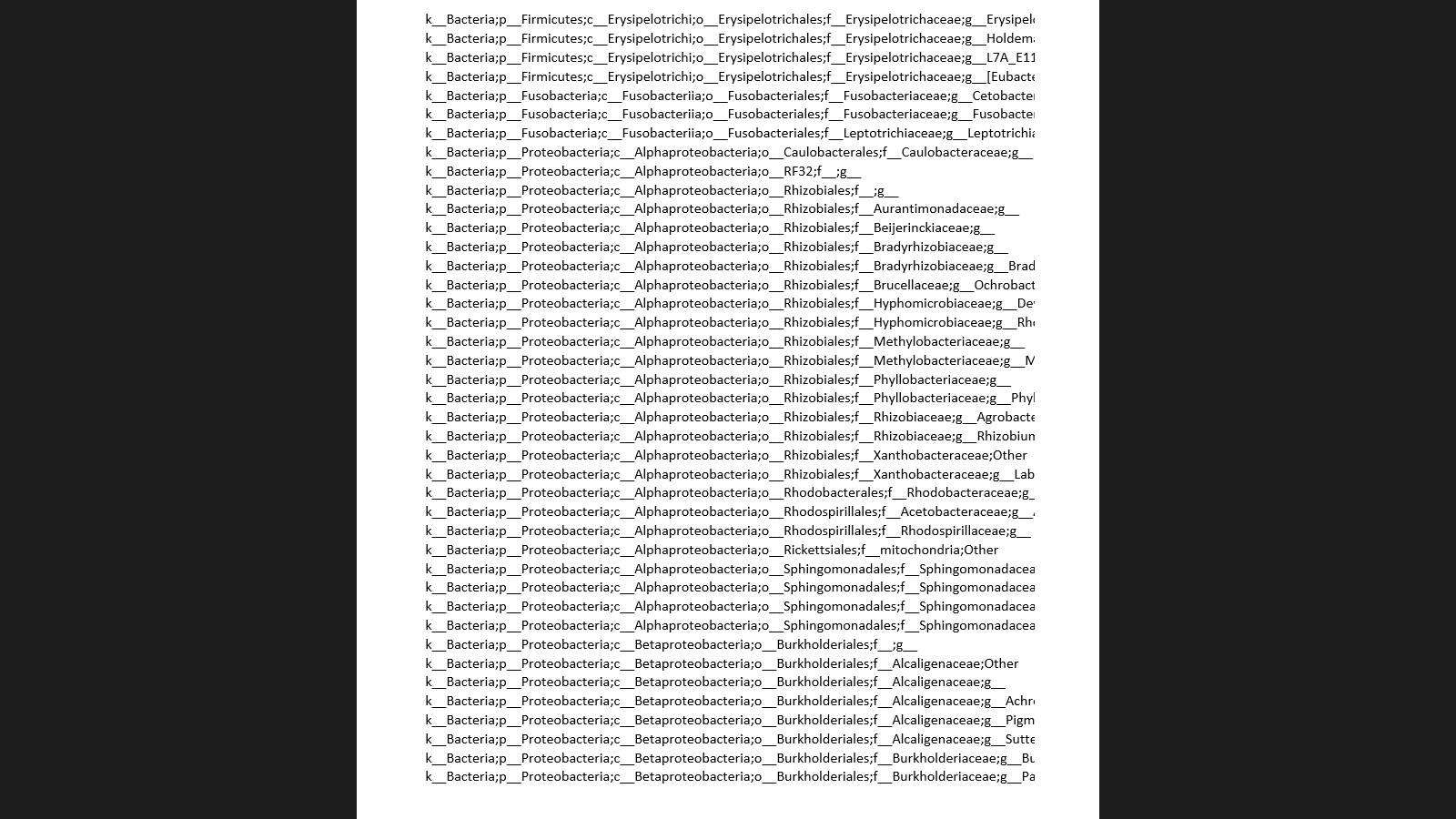


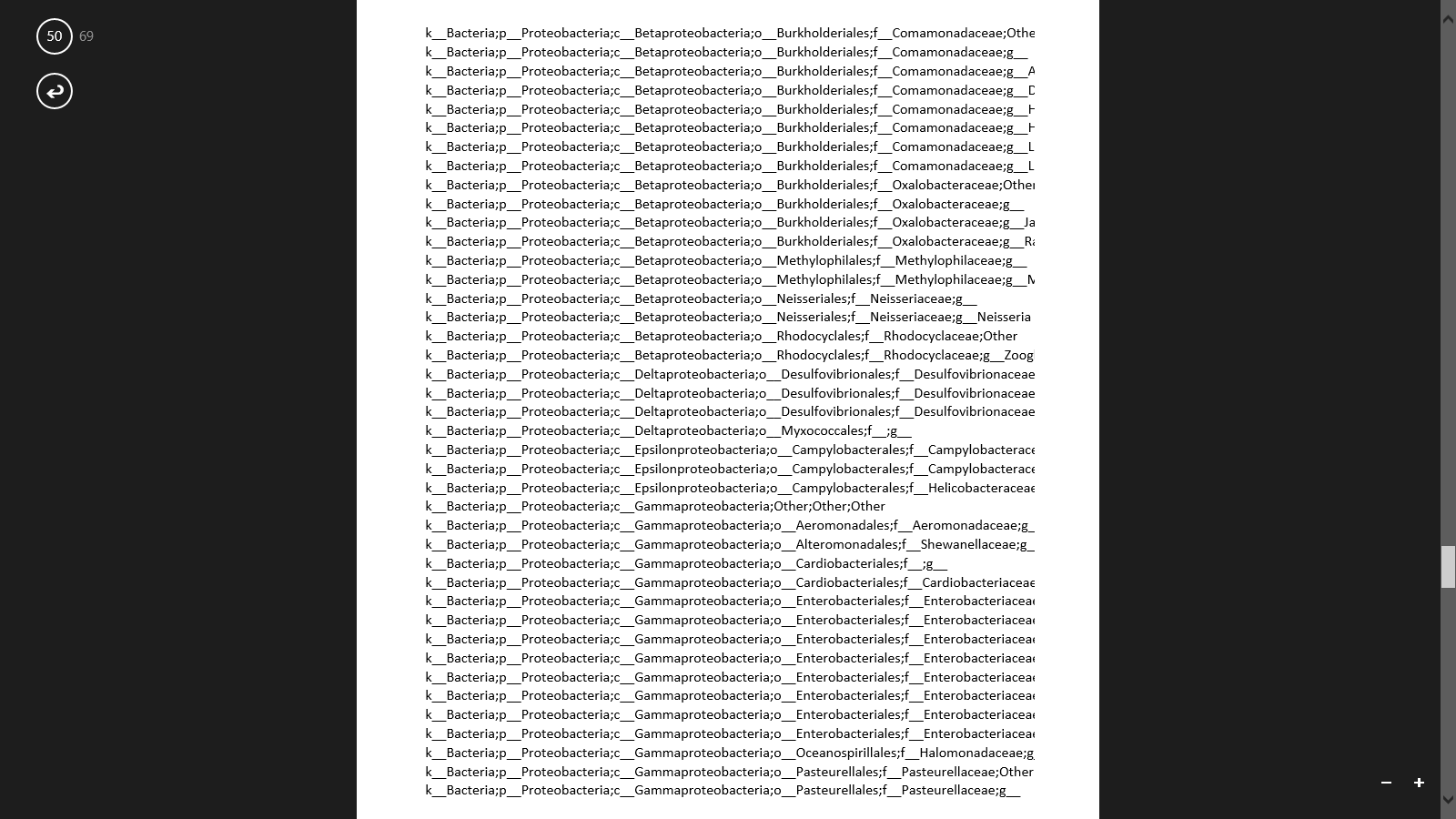


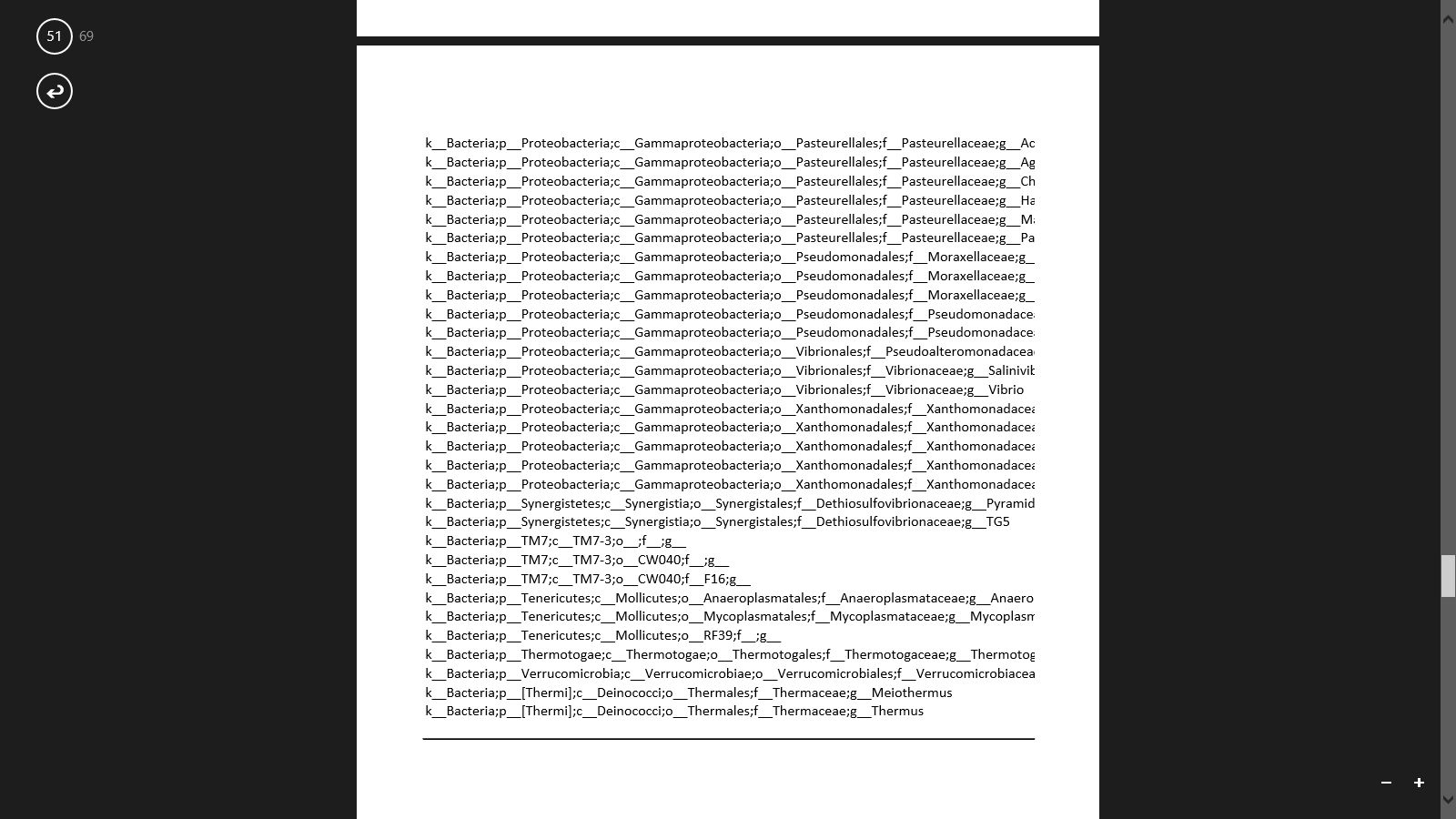


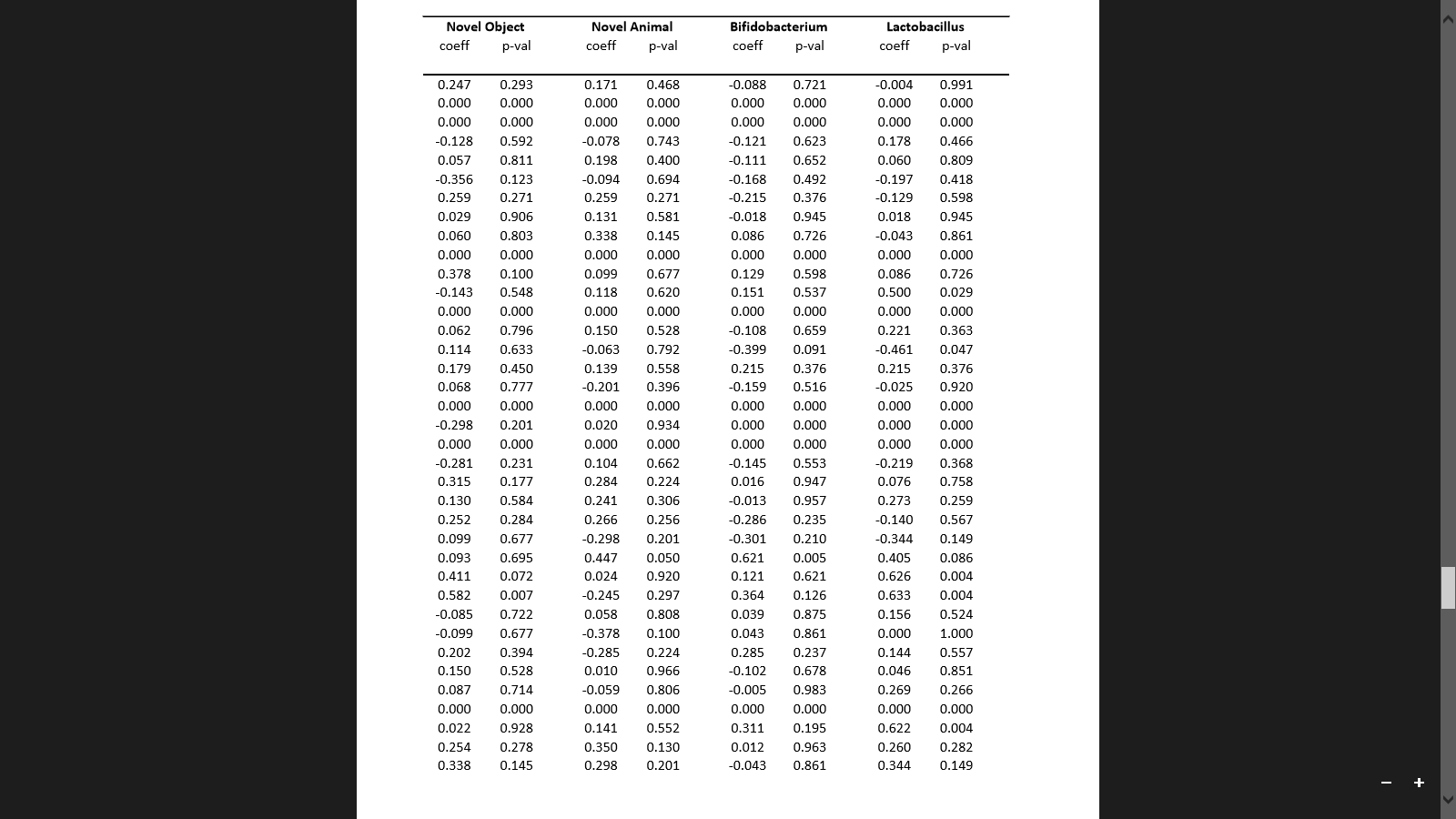


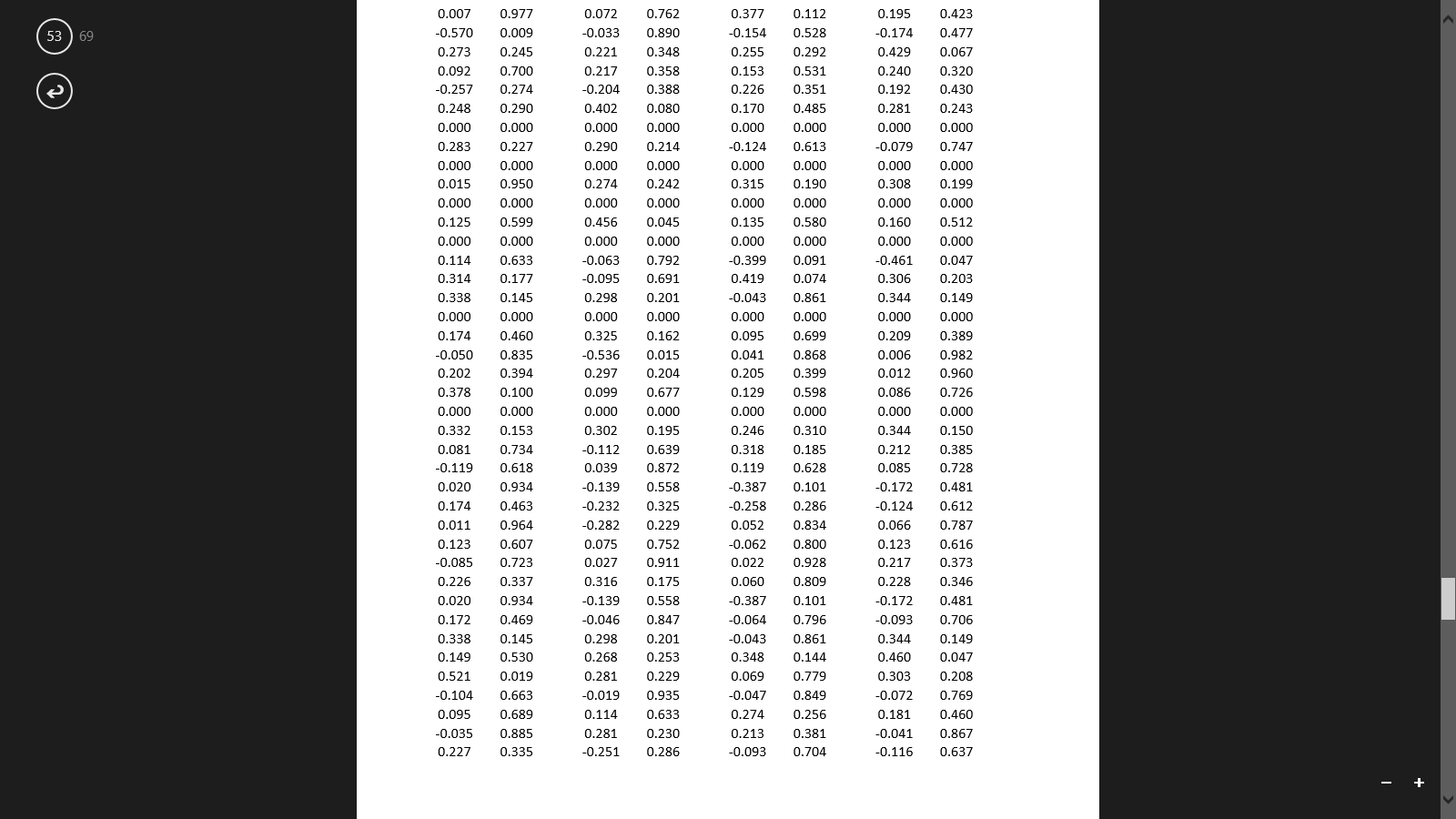


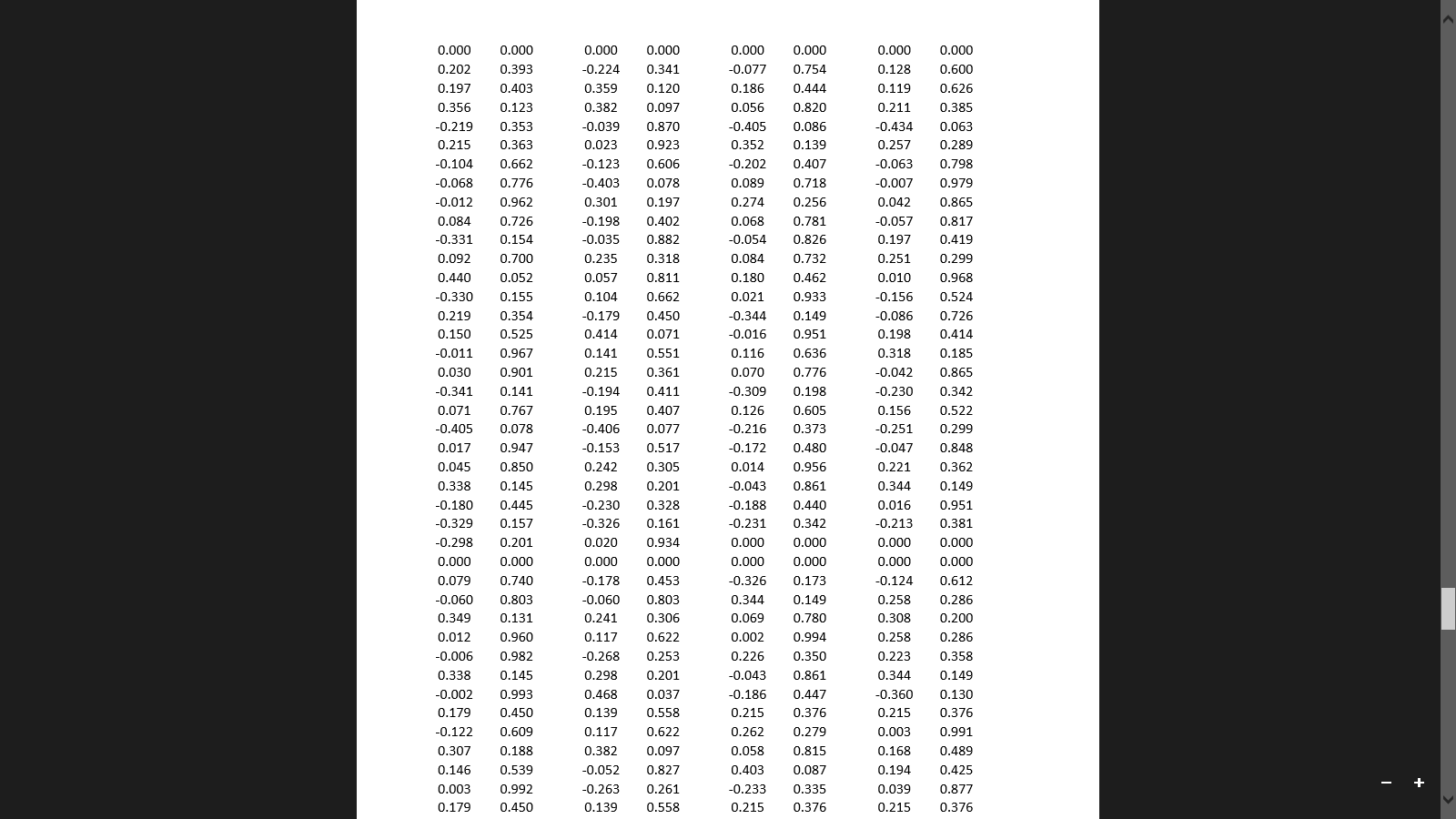


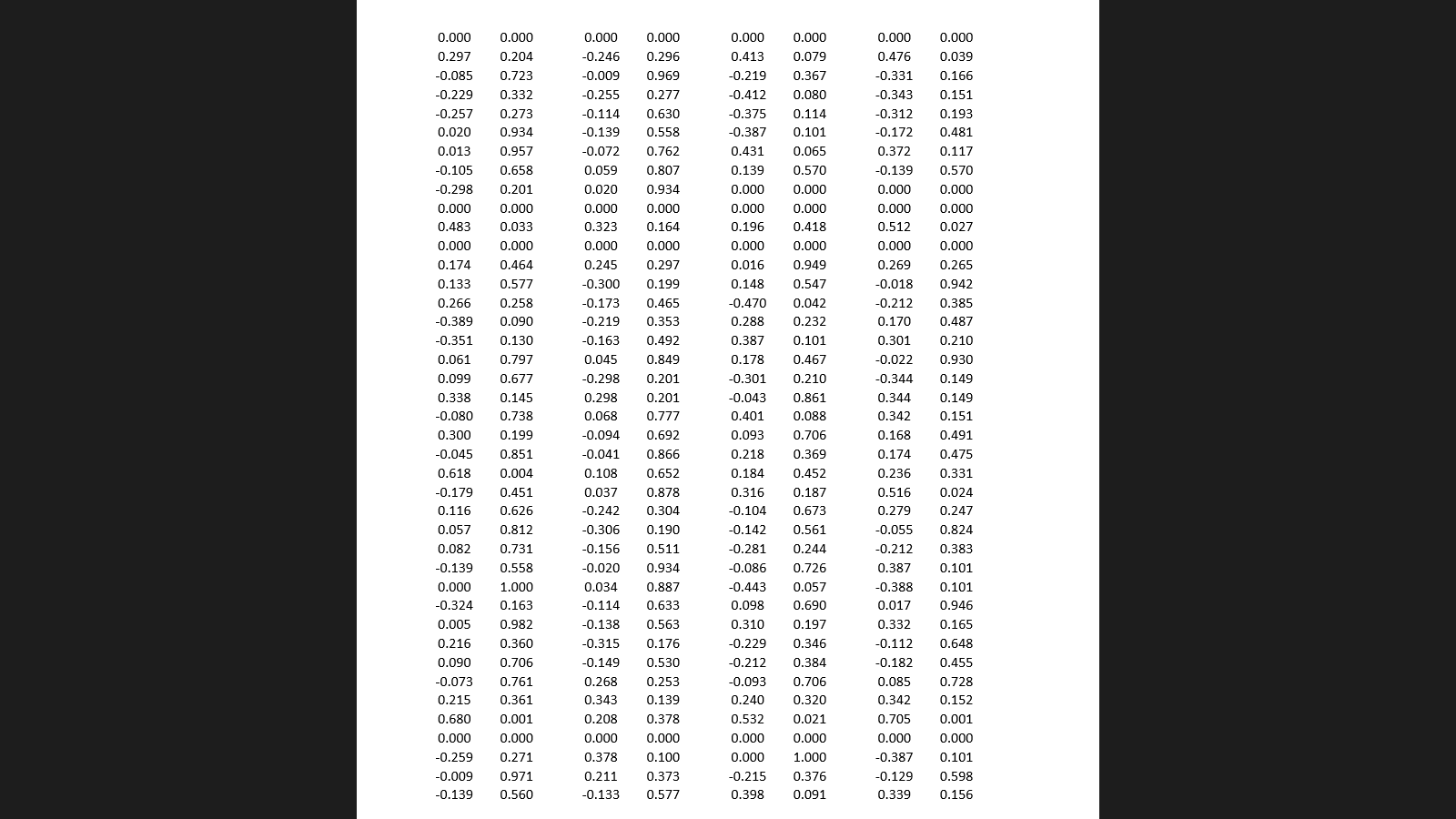


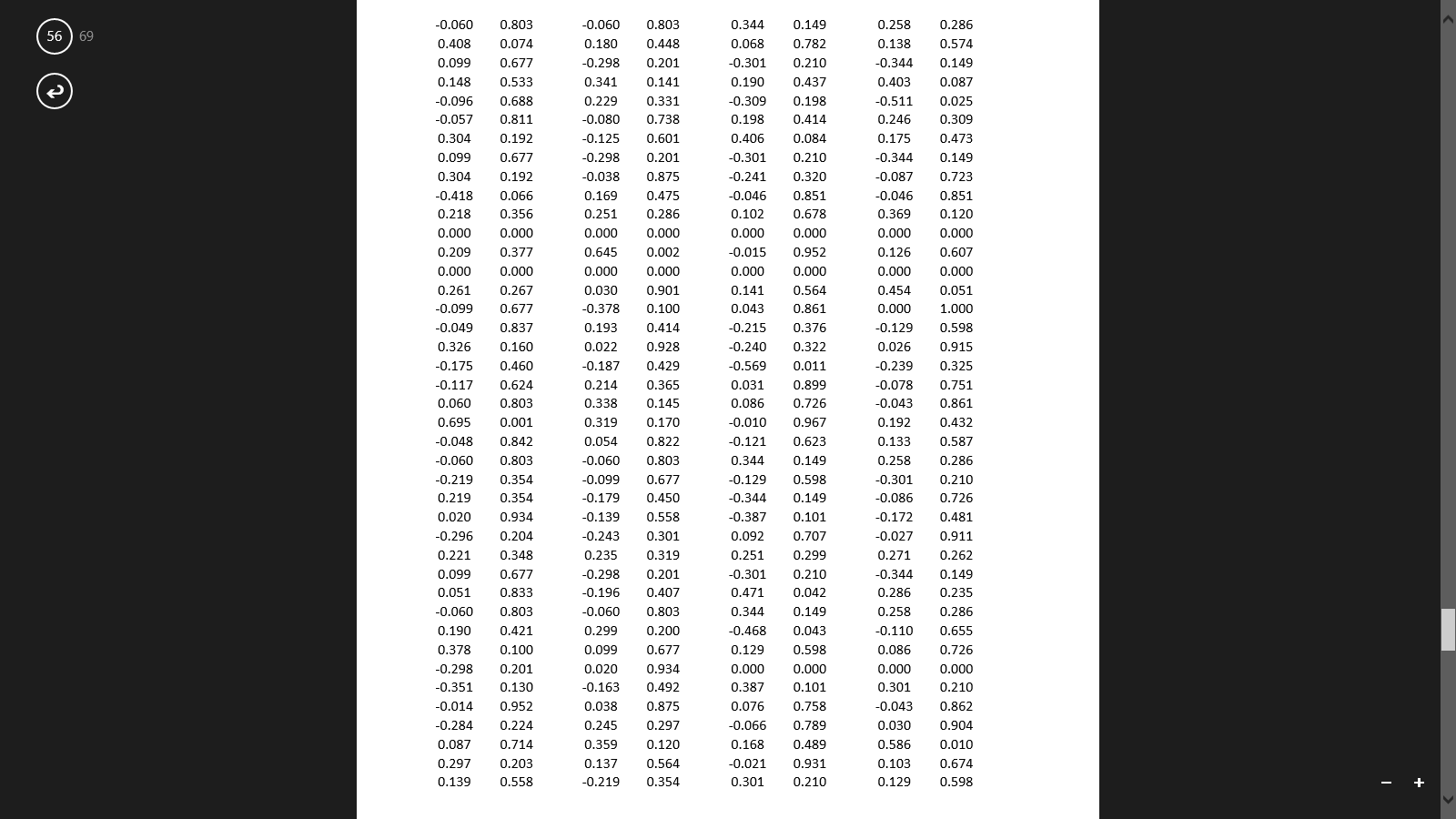


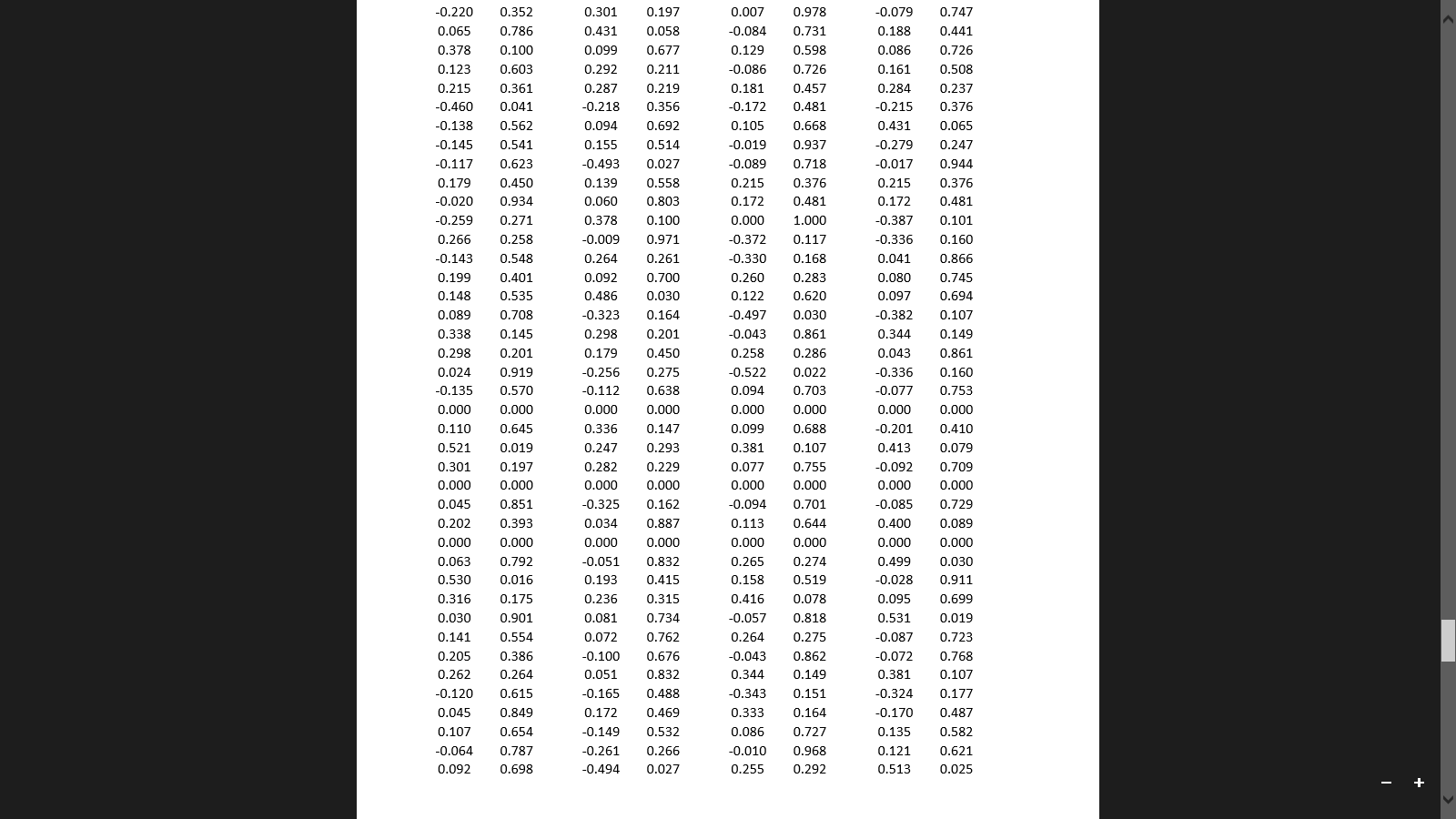


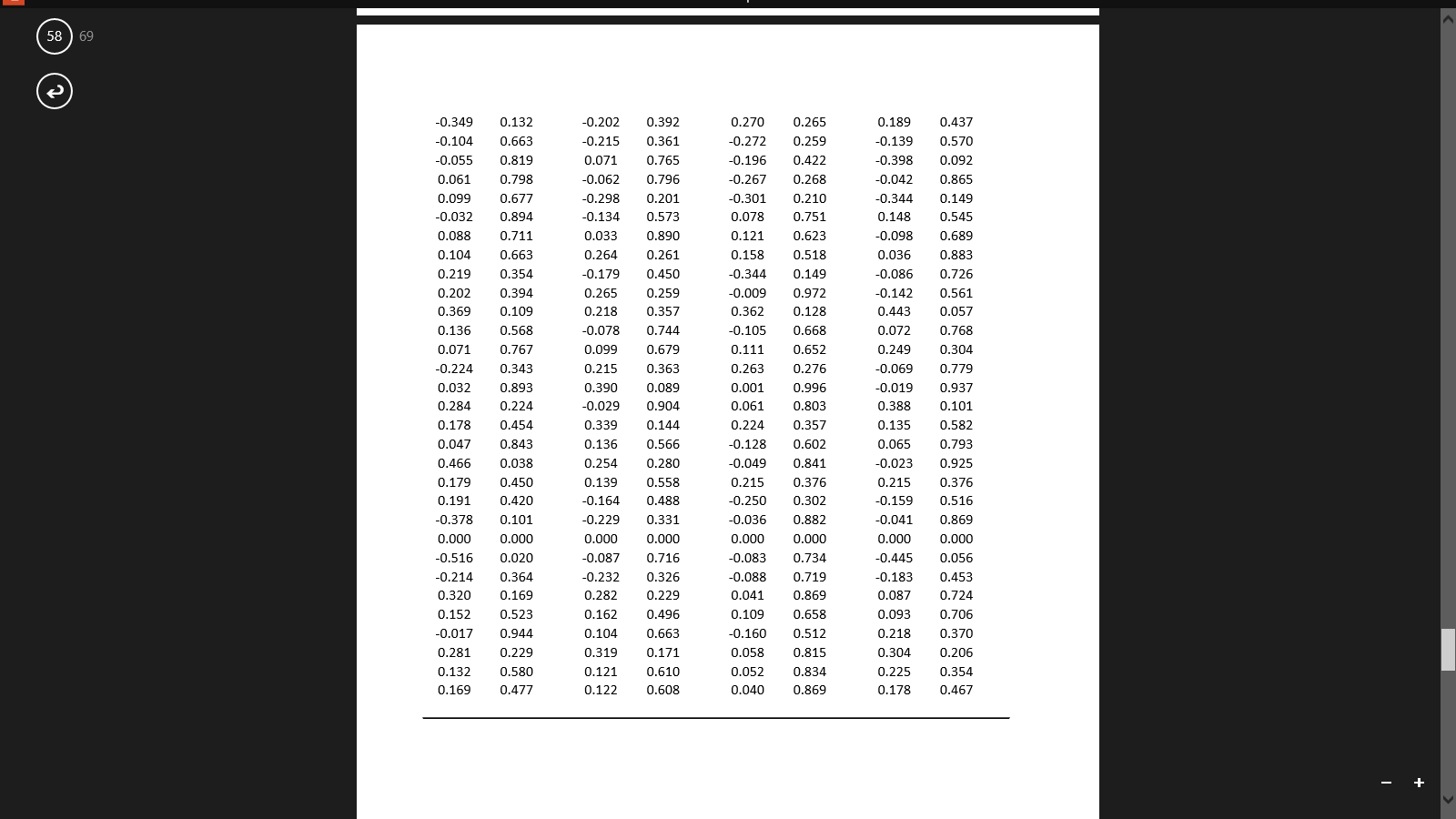


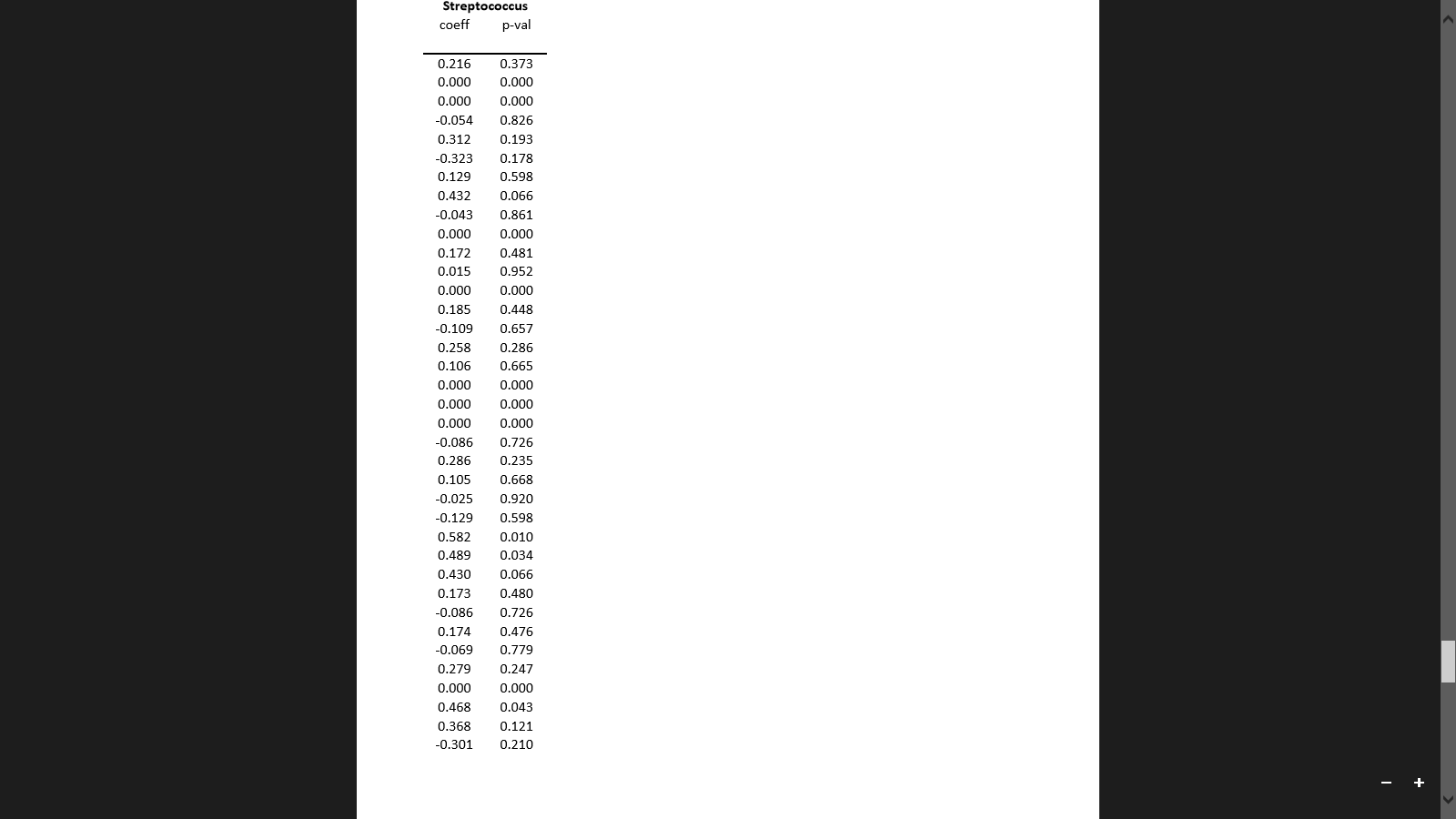


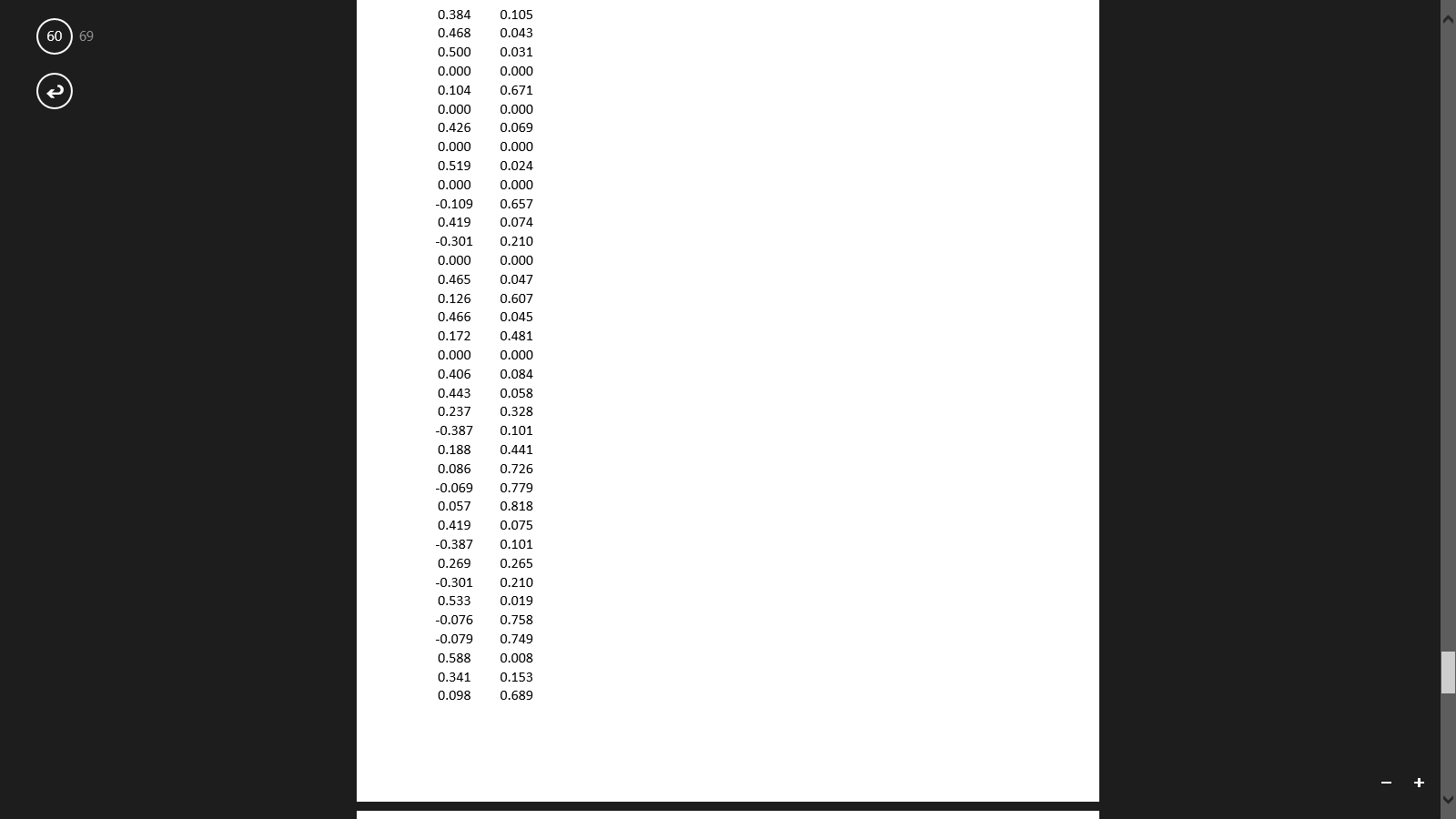


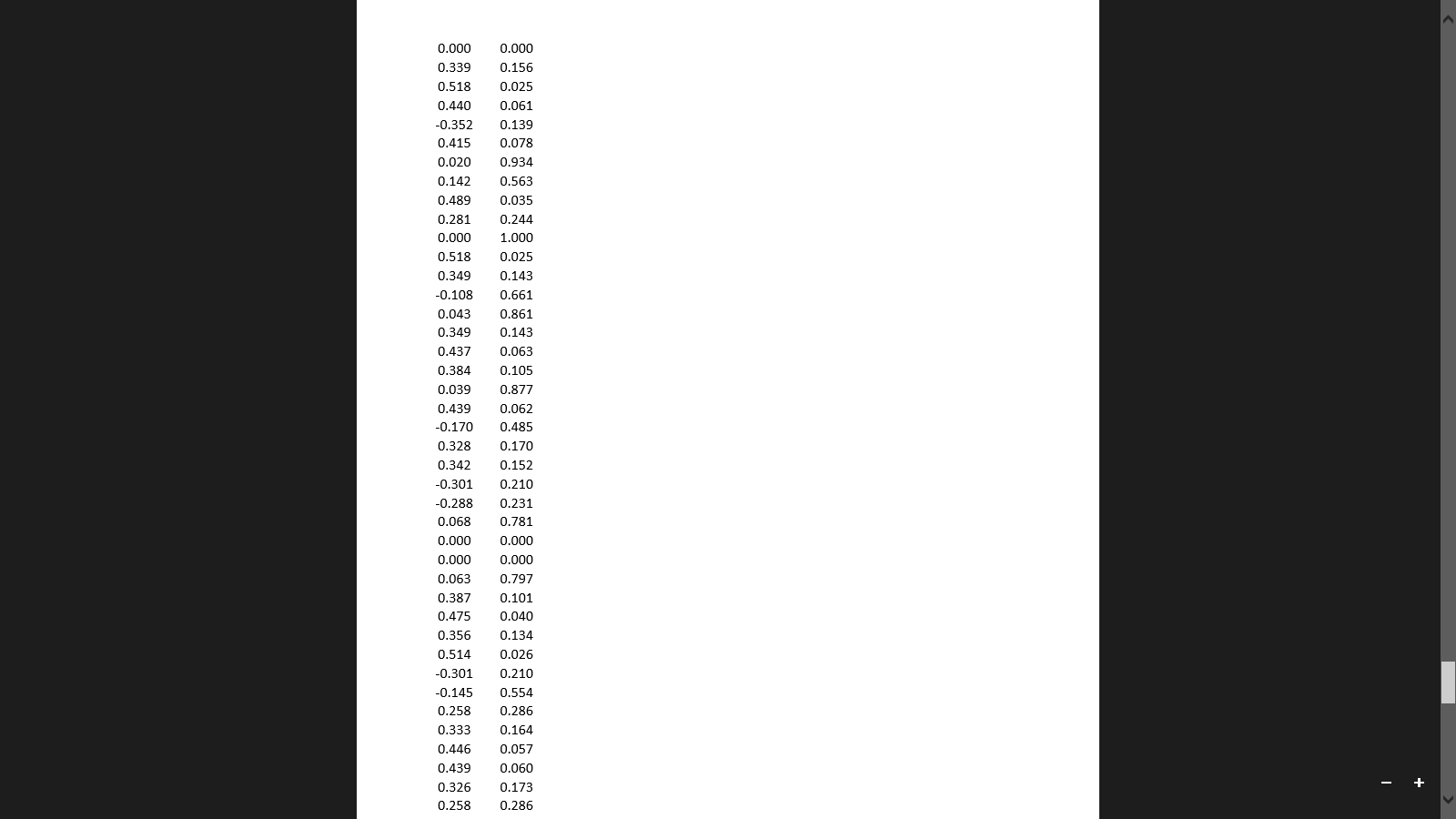


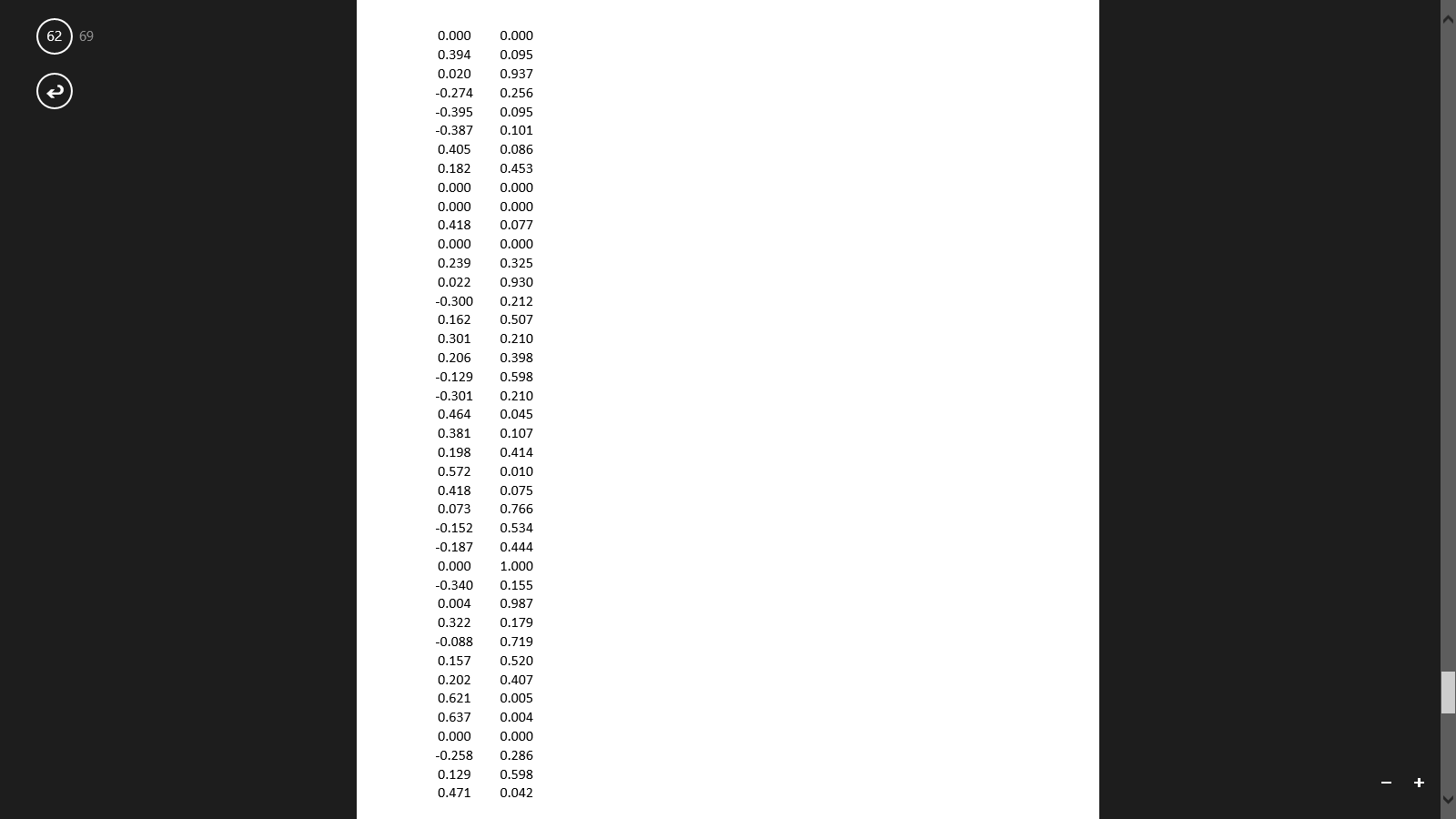


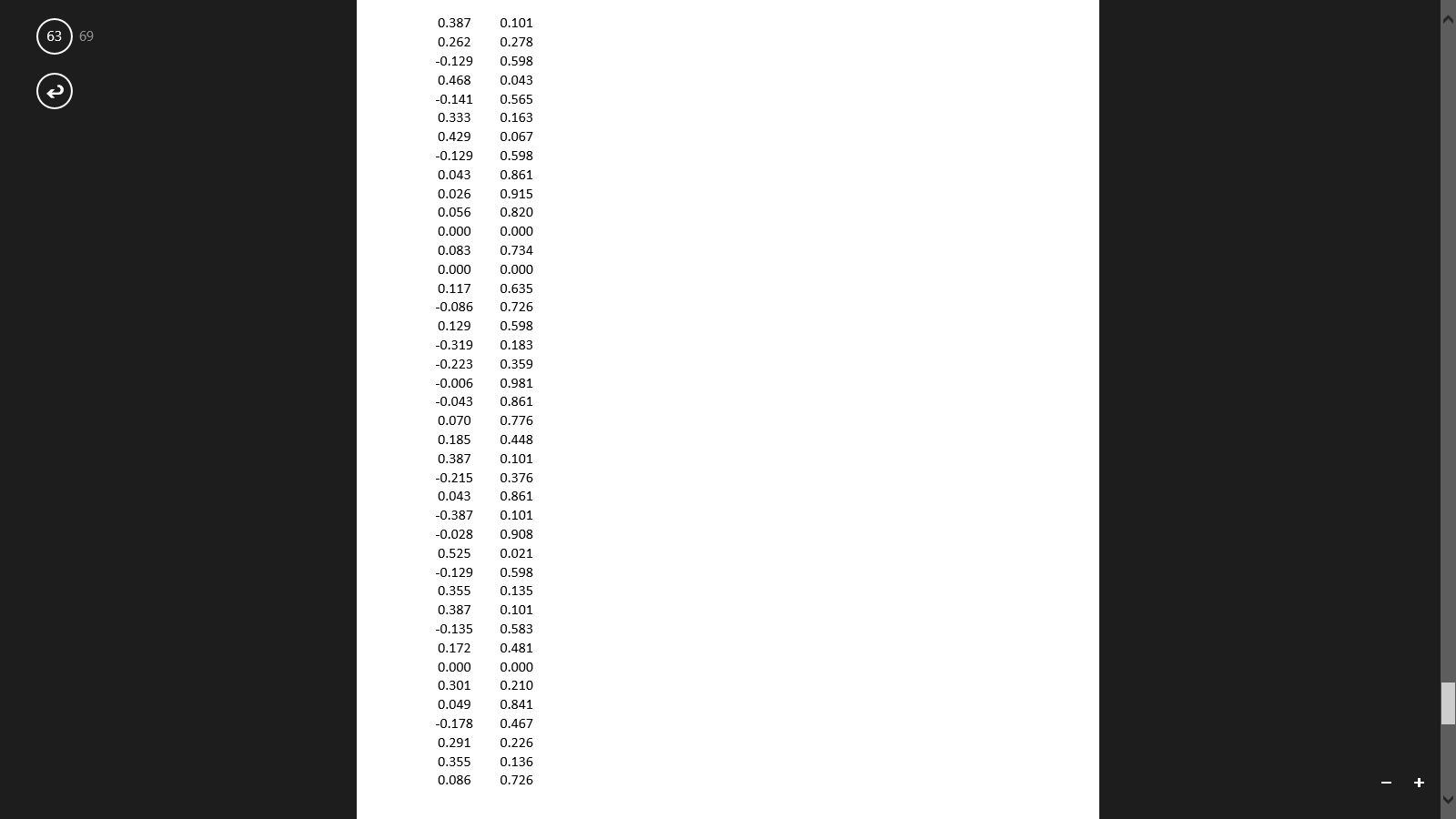


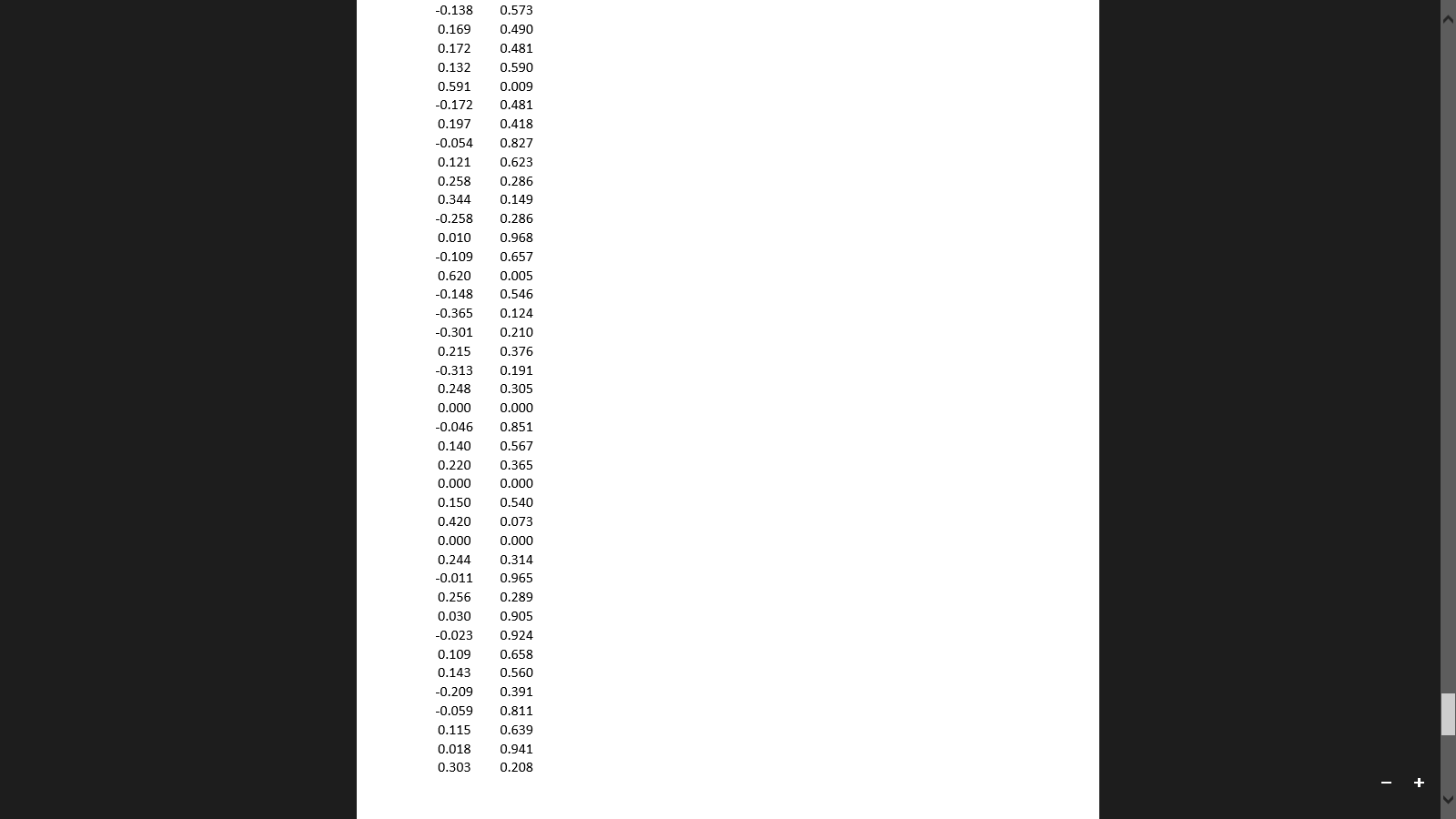
\


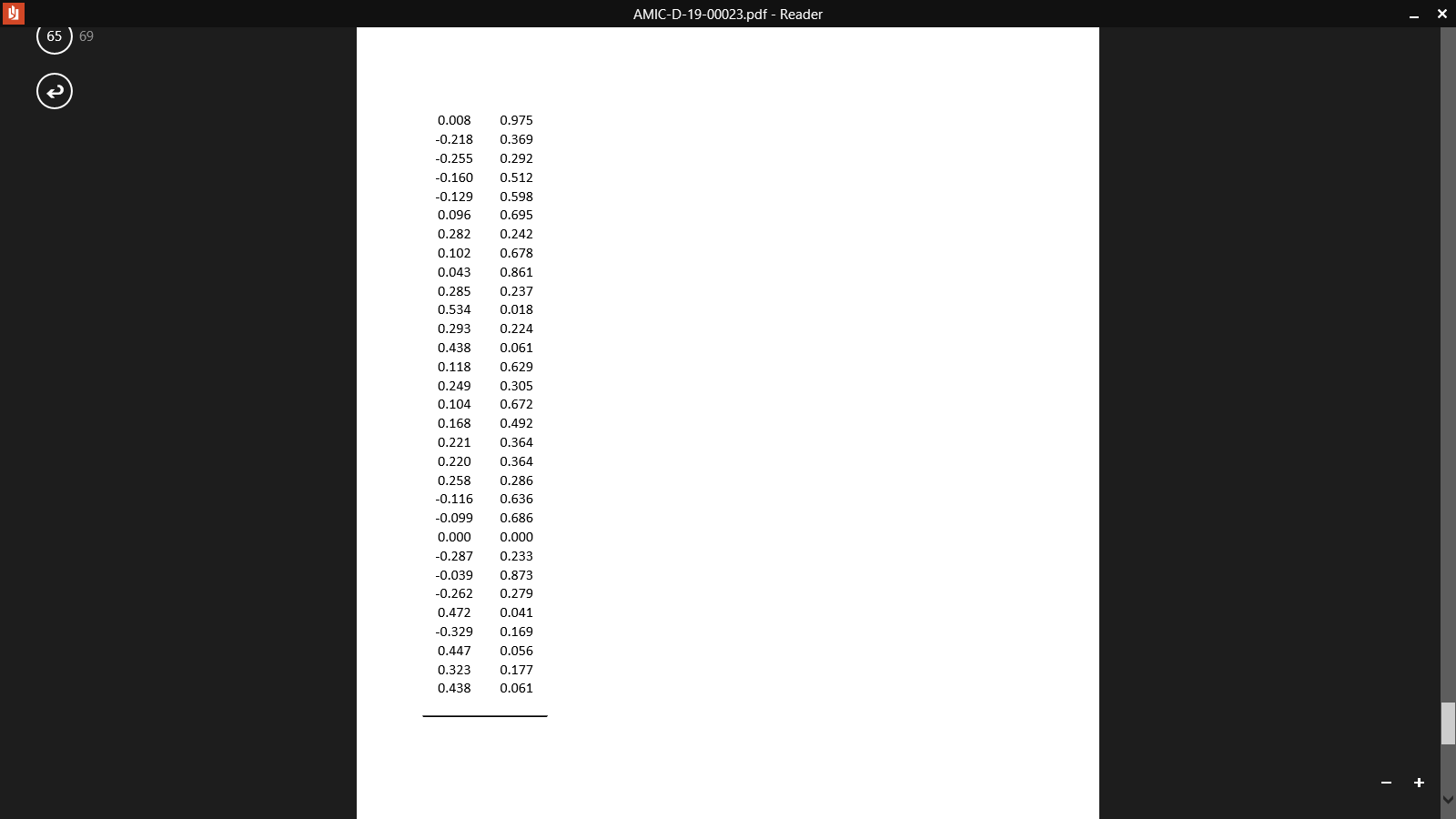

Supplement: Supplementary file 1 — Additional file 1. [file 42523_2020_30_MOESM1_ESM.docx]
